# A transient helix in the disordered region of dynein light intermediate chain links the motor to structurally diverse adaptors for cargo transport

**DOI:** 10.1371/journal.pbio.3000100

**Published:** 2019-01-07

**Authors:** Ricardo Celestino, Morkos A. Henen, José B. Gama, Cátia Carvalho, Maxwell McCabe, Daniel J. Barbosa, Alexandra Born, Parker J. Nichols, Ana X. Carvalho, Reto Gassmann, Beat Vögeli

**Affiliations:** 1 Instituto de Biologia Molecular e Celular (IBMC), Universidade do Porto, Porto, Portugal; 2 Instituto de Investigação e Inovação em Saúde (i3S), Universidade do Porto, Porto, Portugal; 3 Department of Biochemistry and Molecular Genetics, University of Colorado Denver, Anschutz Medical Campus, Aurora, Colorado, United States of America; 4 Faculty of Pharmacy, Mansoura University, Mansoura, Egypt; Johns Hopkins University, UNITED STATES

## Abstract

All animal cells use the motor cytoplasmic dynein 1 (dynein) to transport diverse cargo toward microtubule minus ends and to organize and position microtubule arrays such as the mitotic spindle. Cargo-specific adaptors engage with dynein to recruit and activate the motor, but the molecular mechanisms remain incompletely understood. Here, we use structural and dynamic nuclear magnetic resonance (NMR) analysis to demonstrate that the C-terminal region of human dynein light intermediate chain 1 (LIC1) is intrinsically disordered and contains two short conserved segments with helical propensity. NMR titration experiments reveal that the first helical segment (helix 1) constitutes the main interaction site for the adaptors Spindly (SPDL1), bicaudal D homolog 2 (BICD2), and Hook homolog 3 (HOOK3). In vitro binding assays show that helix 1, but not helix 2, is essential in both LIC1 and LIC2 for binding to SPDL1, BICD2, HOOK3, RAB-interacting lysosomal protein (RILP), RAB11 family-interacting protein 3 (RAB11FIP3), ninein (NIN), and trafficking kinesin-binding protein 1 (TRAK1). Helix 1 is sufficient to bind RILP, whereas other adaptors require additional segments preceding helix 1 for efficient binding. Point mutations in the C-terminal helix 1 of *Caenorhabditis elegans* LIC, introduced by genome editing, severely affect development, locomotion, and life span of the animal and disrupt the distribution and transport kinetics of membrane cargo in axons of mechanosensory neurons, identical to what is observed when the entire LIC C-terminal region is deleted. Deletion of the C-terminal helix 2 delays dynein-dependent spindle positioning in the one-cell embryo but overall does not significantly perturb dynein function. We conclude that helix 1 in the intrinsically disordered region of LIC provides a conserved link between dynein and structurally diverse cargo adaptor families that is critical for dynein function in vivo.

## Abstract 2

A highly conserved mechanism links the microtubule minus end–directed motor dynein to structurally diverse cargo adaptors through its light intermediate chain; this interaction is crucial for dynein function in vivo.

## Author summary

The large size and complex organization of animal cells make the correct and efficient distribution of intracellular content a challenge. The solution is to use motor proteins, which harness energy from ATP hydrolysis to walk along actin filaments or microtubules, for directional transport of cargo. The multi-subunit motor cytoplasmic dynein 1 (dynein) is responsible for transport directed toward the minus ends of microtubules. An important question is how dynein is recruited to its diverse cargo, which includes organelles such as endosomes and mitochondria, proteins, and mRNA. In this study, we use nuclear magnetic resonance spectroscopy to show that the light intermediate chain (LIC) subunit of human dynein uses a short helix in its disordered C-terminal region to bind structurally distinct adaptor proteins that connect the motor to specific cargo. We then use genome editing in the animal model *C*. *elegans* to demonstrate the functional relevance of the C-terminal LIC helix for dynein-dependent cargo transport in neurons. Thus, dynein recruitment to cargo involves a highly conserved interaction between LIC and adaptor proteins.

## Introduction

Microtubule-based cargo transport and force production are critical for a wide range of cellular and developmental processes. In animal cells, the 1.4-MDa complex cytoplasmic dynein 1 (dynein) is the major molecular motor with motility directed toward microtubule minus ends. Dynein-driven cargo transport is particularly crucial in highly polarized cells such as neurons. In axons, whose microtubule plus ends are uniformly oriented toward the axonal tip, dynein is responsible for the retrograde transport of diverse vesicle and organelle cargo toward the cell body. Mutations in dynein that alter axonal transport kinetics have been linked to a variety of nervous system disorders, including spinal muscular atrophy, motor neuron disease, Perry syndrome, and Charcot-Marie-Tooth 2 disease [1,2]. In addition to transporting cargo, dynein can exert pulling forces on microtubules when the motor is stably anchored at subcellular sites such as the cell cortex. A striking example of dynein-dependent force production occurs during mitosis, when cortically localized dynein pulls on astral microtubules to position and orient the bipolar spindle, which in turn defines the axis along which the cell will divide. Dynein's functional versatility implies tight regulation of localization and motor activity, the molecular basis of which has only recently begun to be understood.

Dynein is a 12-subunit complex consisting of a dimerized heavy chain (HC) with a C-terminal motor domain and two copies each of five accessory chains that bind along the HC N-terminal tail: dynein intermediate chain (IC), light intermediate chain (LIC), and three types of light chain (LC). In vivo, dynein function requires the cofactor dynactin, which is itself a 1.1-MDa complex built around a short filament of actin-related protein 1 (ARP1) [3]. In recent years, a number of coiled-coil proteins, referred to as activating adaptors [4], have been shown to recruit dynein to cargo while simultaneously promoting the association of dynein with dynactin [5,6]. Dynein, dynactin, and the N-terminal coiled-coil region of activating adaptors form a stable three-way assembly capable of highly processive motility in vitro [7,8], whereas the C-terminal region of adaptors links to cargo [4]. Cryo–electron microscopy (EM) studies with the adaptors bicaudal D homolog 2 (BICD2), BICD-related protein 1 (BICDR1), and Hook homolog 3 (HOOK3) have revealed the molecular arrangement within the dynein-dynactin-adaptor assembly [3,9–11]: the adaptor coiled-coil region binds along the length of the dynactin ARP1 filament with the adaptor N terminus located at the filament's barbed end, and the N-terminal tail of dynein HC makes contact with both the ARP1 filament and the coiled-coil region of the adaptor.

An additional contact between dynein and adaptors involves the C-terminal region of the LIC subunit (LIC-C) [12,13]. Whereas the highly conserved N-terminal GTPase-like domain of LIC binds tightly to the HC [12,14], the LIC-C sequence is more divergent and predicted to be disordered. Vertebrates possess two genes for LIC (LIC1 and LIC2), which may specify distinct dynein populations. Adaptors known to bind LIC1-C include BICD2 and Spindly (SPDL1), which are likely related [15,16], as well as the structurally distinct adaptors HOOK3, RAB11 family-interacting protein 3 (RAB11FIP3; hereafter referred to as FIP3), and RAB-interacting lysosomal protein (RILP) [12,17]. Whether LIC2-C also interacts with these adaptors has not been examined. SPDL1/BICD2 and HOOK3 bind to LIC1-C through a motif in their first coiled-coil segment (the CC1 box) and the N-terminal Hook domain, respectively, and point mutations in these adaptors that abrogate binding to LIC1-C compromise the formation of the dynein-dynactin-adaptor assembly [16–18]. Consequently, HOOK3 mutants that fail to bind LIC1-C do not support processive dynein runs in vitro [17]. A mutation in the CC1 box of *Drosophila melanogaster* BicD causes a hypomorphic loss-of-function phenotype [19], indicating that the BicD-LIC interaction is functionally relevant in vivo. Numerous other loss-of-function studies have implicated LIC in many dynein-dependent processes, including mitosis and retrograde cargo transport in axons. However, the extent to which LIC loss-of-function phenotypes reflect an important role for LIC-C is less clear, as the dynein complex becomes destabilized when LIC is absent in *D*. *melanogaster* and *Aspergillus nidulans* [20,21], as well as in LIC1-deficient mice [22]. Indeed, biochemical analysis indicates that the N-terminal LIC domain plays an important structural role within the dynein complex [14,23]. Thus, although LIC is clearly essential for dynein function in vivo, the specific contributions of LIC-C remain to be determined.

Here, we dissect the interaction between LIC-C and dynein adaptors in vitro and in the animal model *C*. *elegans*. Nuclear magnetic resonance (NMR) analysis shows that human LIC1-C is intrinsically disordered and possesses two short segments with helical propensity. In agreement with a recent report [13], we show that helix 1 of LIC1-C is essential for binding to BICD2, SPDL1, and HOOK3, and we extend this finding to the adaptors FIP3, RILP, ninein (NIN), and trafficking kinesin-binding protein 1 (TRAK1), as well as to LIC2-C. Finally, we show that LIC-C mutants generated by genome editing in *C*. *elegans* have major defects in postembryonic cell division and retrograde axonal cargo transport, demonstrating the crucial importance of LIC-C helix 1 for dynein function in vivo.

## Results

### The C-terminal region of LIC1 is intrinsically disordered and contains two short segments with α-helical propensity

**Fig 1:**
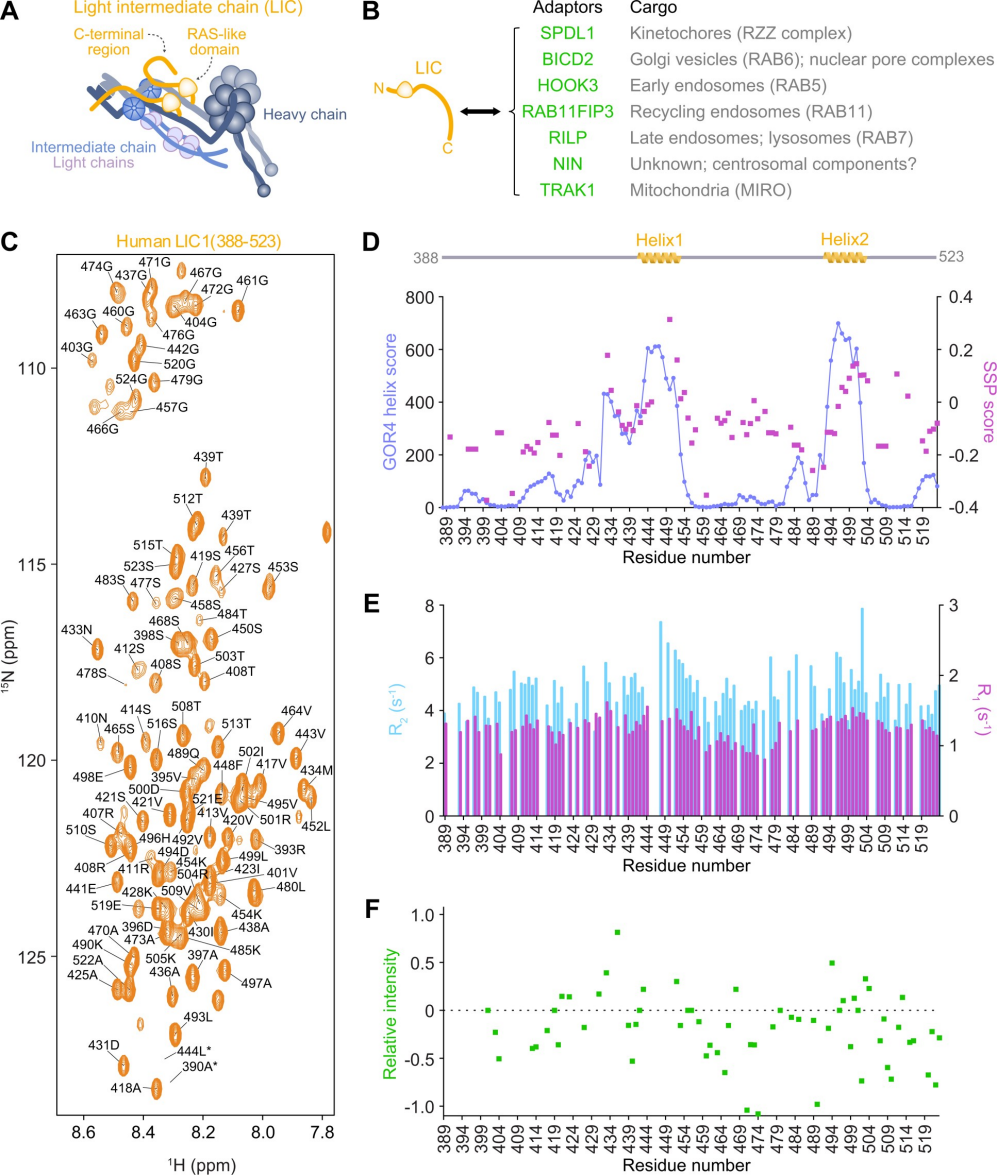
LIC1(388–523) is intrinsically disordered and contains two short segments with α-helical propensity. (A) Subunit composition of dynein. The N-terminal RAS-like domain of the LIC binds to the N-terminal tail of the HC, and the flexible LIC C-terminal region interacts with cargo adaptor proteins, as illustrated in (B). (B) Cargo adaptor proteins examined in this study that bind the C-terminal region of dynein LIC. (C) ^15^N-^1^H HSQC spectrum of human LIC1(388–523) with backbone NH assignments. The spectra were acquired at 25°C and 900 MHz. Labels marked with an asterisk indicate peaks with heights below the cutoff contour level. (D) SSP scores for LIC1(388–523) calculated using ^1^H^N^, ^15^N, CO, CA, and CB chemical shifts for each assigned residue. “+1” indicates a fully formed α helix, “-1” indicates a fully formed β sheet, and “0” indicates disorder. GOR4 helix prediction scores are also shown. Values from 0 to 1,000 reflect absence of a fully formed α-helical structure. Scores obtained for residues 442–453 and 493–502 indicate transient α helix formation. (E) ^15^N relaxation measurements for LIC1(388–523). *R*_1_ and *R*_2_ relaxation rate constants at 700 MHz are shown for each assigned residue. (F) ^15^N-^1^H heteronuclear NOEs of LIC1(388–523) probing amide bond vector motion. Relaxation rate constants (E) and NOEs (F) are indicative of intrinsic structural disorder, and larger values indicate more restricted sub-nanosecond motion, which are observed for the helical regions. Underlying data for Fig 1 can be found in S1 Data. BICD2, bicaudal D homolog 2; dynein, cytoplasmic dynein 1; HC, heavy chain; HOOK3, Hook homolog 3; HSQC, heteronuclear single quantum coherence; LIC, light intermediate chain; MIRO, mitochondrial Rho; NIN, ninein; NOE, nuclear Overhauser enhancement; ppm, parts per million; RAB11FIP3, RAB11 family-interacting protein 3; RILP, RAB-interacting lysosomal protein; RZZ, ROD-ZW10-ZWILCH; SPDL1, Spindly; SSP, secondary structure propensity; TRAK1, trafficking kinesin-binding protein 1.

To gain mechanistic insight into how LIC-C interacts with dynein adaptors that are diverse in structure and function (Fig 1A and 1B, S1 Fig), we first characterized LIC1-C (residues 388–523) by NMR spectroscopy. The ^15^N-^1^H heteronuclear single quantum coherence (HSQC) spectrum of LIC1-C at 25°C showed a narrow 7.8 to 8.6 parts per million (ppm) amide-proton chemical shift range, indicating a predominance of structural disorder (Fig 1C). Near-complete backbone resonance assignment was achieved (104 out of the 117 nonproline residues; Fig 1C, S2 Fig). An overlay of ^15^N-^1^H HSQC spectra of two smaller constructs, consisting of residues 388–471 and 472–523, reproduced the spectrum obtained from the entire LIC1-C (S2A Fig). This finding indicates that there are no long-range interactions between N- and C-terminal segments of LIC1-C.

To assess residual secondary structure in LIC1-C, we computed the secondary structure propensity (SSP) score developed by Forman-Kay and colleagues [24]. The method combines different backbone chemical shifts into a single residue–specific SSP score. The results closely matched those obtained from the GOR4 secondary structure prediction program [25] (Fig 1D, S1 Data). An SSP score of +1 and −1 indicates a fully formed α helix and β sheet, respectively. The SSP scores for LIC1-C residues with predicted α-helical propensity (residues 444–454 and 497–504) were positive values up to 0.35. However, assuming a linear relationship between the score and the population, helical structure was only transiently sampled at about 20%. These two segments of LIC1-C, which are highly conserved (S1A Fig), will be referred to as helix 1 and helix 2, respectively. The remaining segments scored mostly slightly negative SSP values and GOR4 values around zero, indicating complete structural disorder (note that β sheet and random coil have both extended secondary structure with similar scores in practice).

To characterize the dynamic properties of LIC1-C, we performed ^15^N NMR relaxation measurements (Fig 1E, S3 Fig). The relaxation rates are sensitive probes of the amplitudes and timescales of residue-specific structural fluctuations. The longitudinal relaxation rates *R*_1_, which report on motion faster than nanoseconds, were 1.2 s^−1^ on average. Although such a value is not compatible with a globular protein, it is indicative of an intrinsically disordered protein that undergoes extensive local reorientation. Residues 441–456 and 492–508 had larger *R*_1_ values, which are suggestive of increased structural order of the residues with α-helical propensity. The residues between helices 1 and 2 had consistently lower-than-average *R*_1_ values, indicating a highly flexible interhelical linker.

The fast dynamics inferred from *R*_1_ measurements reduce the transverse relaxation rates *R*_2_ typically observed for folded proteins, but in addition, potential motion slower than nanoseconds might increase them. Whereas the measured *R*_2_ average of 4.7 s^−1^ is again only reconcilable with high structural disorder, the helical regions showed larger values in line with partial helix formation (Fig 1E). Since the *R*_1_ values suggest less flexibility in the helices on the fast timescale, it is likely that the increased *R*_2_ values originate also exclusively from reduced fast-motion amplitudes. In support of this, relaxation dispersion experiments did not indicate any slow micro-millisecond dynamics. The segment with the smallest values was again the interhelical linker. Since slow motion is absent throughout the protein, the ratio *R*_2_/*R*_1_ allowed extraction of an effective residue-specific reorientational tumbling time that is independent of the motional amplitude [26]. The average tumbling time was 4.5 ns, which is much smaller than a typical value expected for a folded protein consisting of approximately 140 residues.

The ^1^H-^15^N nuclear Overhauser enhancement (NOE) is another probe of the amplitudes of motions taking place on the sub-nanosecond timescale (Fig 1F). The values of nearly all residues fall below 0.5, which is again indicative of structural disorder with large amplitudes. In support of the findings described above, the largest values were typically found for the helix 1 and 2 segments, which suggests higher structural order than the other regions.

In conclusion, the relaxation data show that LIC1-C is intrinsically disordered with large amplitudes of motions faster than nanoseconds. In helix 1 and 2, the amplitudes are smaller and the local reorientation slower but still on the fast timescale. These results support the highly transient character of the helices derived from the SSP score.

For independent confirmation of the residual helical propensity in LIC1-C, we collected circular dichroism (CD) spectra of LIC1-C (S4 Fig). Whereas fully formed α-helical proteins show prominent negative bands at 222 and 208 nm and a positive band at 193 nm, disordered proteins have very low ellipticity above 210 nm and negative bands near 195 nm [27]. The spectrum of LIC1-C corresponded largely to structural disorder. However, there were negative values at 208 nm and a negative shoulder at 222 nm. These features are indicative of residual helical structure, in agreement with our findings from NMR spectroscopy. The helical content was maintained over a large temperature range (25, 37, 50, 60, and 70°C), similar to previous reports on other intrinsically disordered proteins [28].

### The C-terminal helix 1 in LIC1 is the main binding site for SPDL1, BICD2, and HOOK3

**Fig 2:**
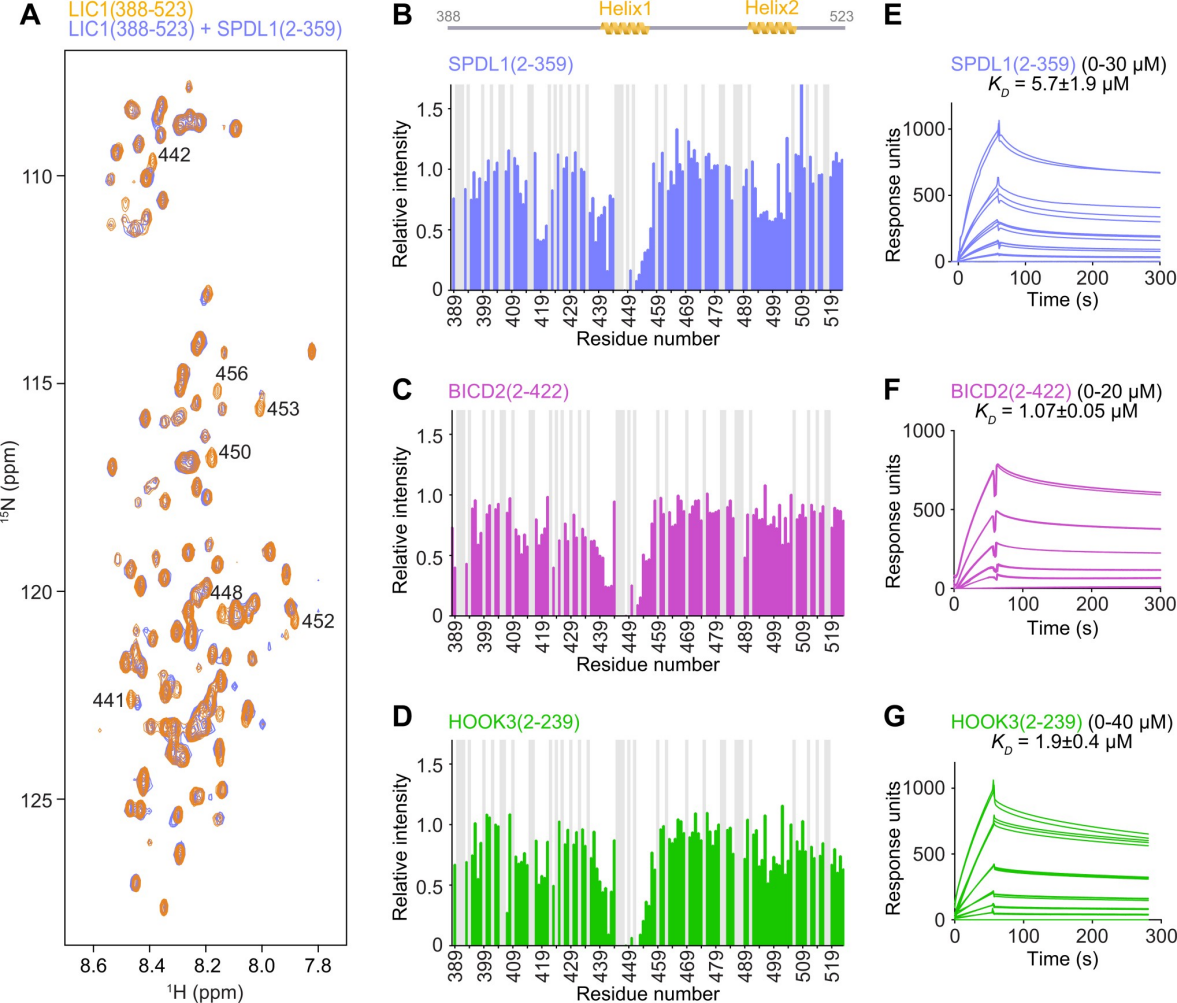
The LIC1(388–523) interaction with the N-terminal regions of SPDL1, BICD2, and HOOK3 at single-residue resolution. (A) Overlay of NMR ^15^N-^1^H HSQC spectra of free ^15^N-labeled LIC1(388–523) (orange) and in a 1:1 mixture with unlabeled SPDL1(2–359) (blue). (B–D) Relative peak intensity quenching in the ^15^N-^1^H HSQC spectra of ^15^N-labeled LIC1(388–523) upon 1:1 titration of unlabeled SPDL1(2–359) (B), BICD2(2–422) (C), and HOOK3(2–239) (D). Gray bars indicate residues for which no ratio could be obtained because of peak overlap or because they were proline residues. (E–G) SPR sensograms from injection of dilution series of SPDL1(2–359) (E), BICD2(2–422) (F), and HOOK3(2–239) (G) over immobilized LIC1(388–523). Fitted *K*_*D*_ values are indicated as mean ± SEM. Underlying data for Fig 2 can be found in S1 Data. BICD2, bicaudal D homolog 2; HOOK3, Hook homolog 3; HSQC, heteronuclear single quantum coherence; LIC1, light intermediate chain 1; NMR, nuclear magnetic resonance; ppm, parts per million; SPDL1, Spindly; SPR, surface plasmon resonance.

To identify adaptor binding sites in LIC1-C, we recorded ^15^N-^1^H HSQC spectra of LIC1-C bound to SPDL1(2–359), BICD2(2–422), and HOOK3(2–239) using molar ratios of 1:0, 1:0.5, and 1:1 (Fig 2A, 2B, 2C and 2D). The spectra obtained from each titration series revealed similar modes of interaction for the three adaptors. Rather than chemical shifts being perturbed, many peaks were significantly attenuated because of binding. This indicates decrease in the local motion of the bound residues; increase of the hydrodynamic radius of the entire complex, resulting in increase in the effective tumbling time; and/or contributions from conformational and chemical exchange [29]. Inspection of the peak height ratios between bound and free LIC1-C showed that the residues affected were mainly located in helix 1 and to a lesser extent in helix 2 (Fig 2B, 2C and 2D). In addition, we observed intensity reduction for the peaks of residues 418–421, which also tend to be conserved (S1A Fig). The quenching of segment 418–421 and helix 2 was more pronounced for SPDL1(2–359) than for BICD2(2–422) or HOOK3(2–239) (Fig 2B, 2C and 2D).

To assess binding affinities between LIC1-C and adaptors, we conducted surface plasmon resonance (SPR) experiments in which LIC1-C::6xHis was immobilized on a nitrilotriacetic acid (NTA) chip. We obtained a *K*_*D*_ of 5.7 ± 1.9 μM for the interaction between LIC1(388–523) and SPDL1(2–359) (Fig 2E). LIC1(388–471), which contains helix 1 but not helix 2, bound to SPDL1(2–359) with the same affinity as LIC1(388–523) (5.40 ± 0.25 μM). Similarly, LIC1(388–523) and LIC1(388–471) bound to BICD2(2–422) with comparable affinity (*K*_*D*_ of 1.07 ± 0.05 μM and 0.7 ± 0.01 μM, respectively) (Fig 2F). We also measured binding to fluorescently labeled LIC1(388–523) in microscale thermophoresis (MST) experiments. We obtained *K*_*D*_ values of 13.1 ± 0.4 μM and 6.0 ± 1.3 μM for the interaction with SPDL1(2–359) and BICD2(2–422), respectively, which is in reasonable agreement with the SPR analysis (S5A and S5B Fig). From SPR and MST experiments with LIC1(388–523) and HOOK3(2–239), we obtained *K*_*D*_ values of 1.9 ± 0.4 μM and 4.0 ± 0.8 μM, respectively (Fig 2G, S5C Fig), which are similar to those obtained with BICD2(2–422). We conclude that SPDL1, BICD2, and HOOK3 bind to LIC1-C with an affinity in the single-digit micromolar range and that an LIC1-C fragment comprising residues 388–471 is sufficient for binding. These results are in agreement with the NMR titration experiments, which showed the most pronounced intensity quenching for helix 1 residues.

### The C-terminal helix 1 in LIC1 and LIC2 is essential for binding to structurally diverse adaptors

**Fig 3:**
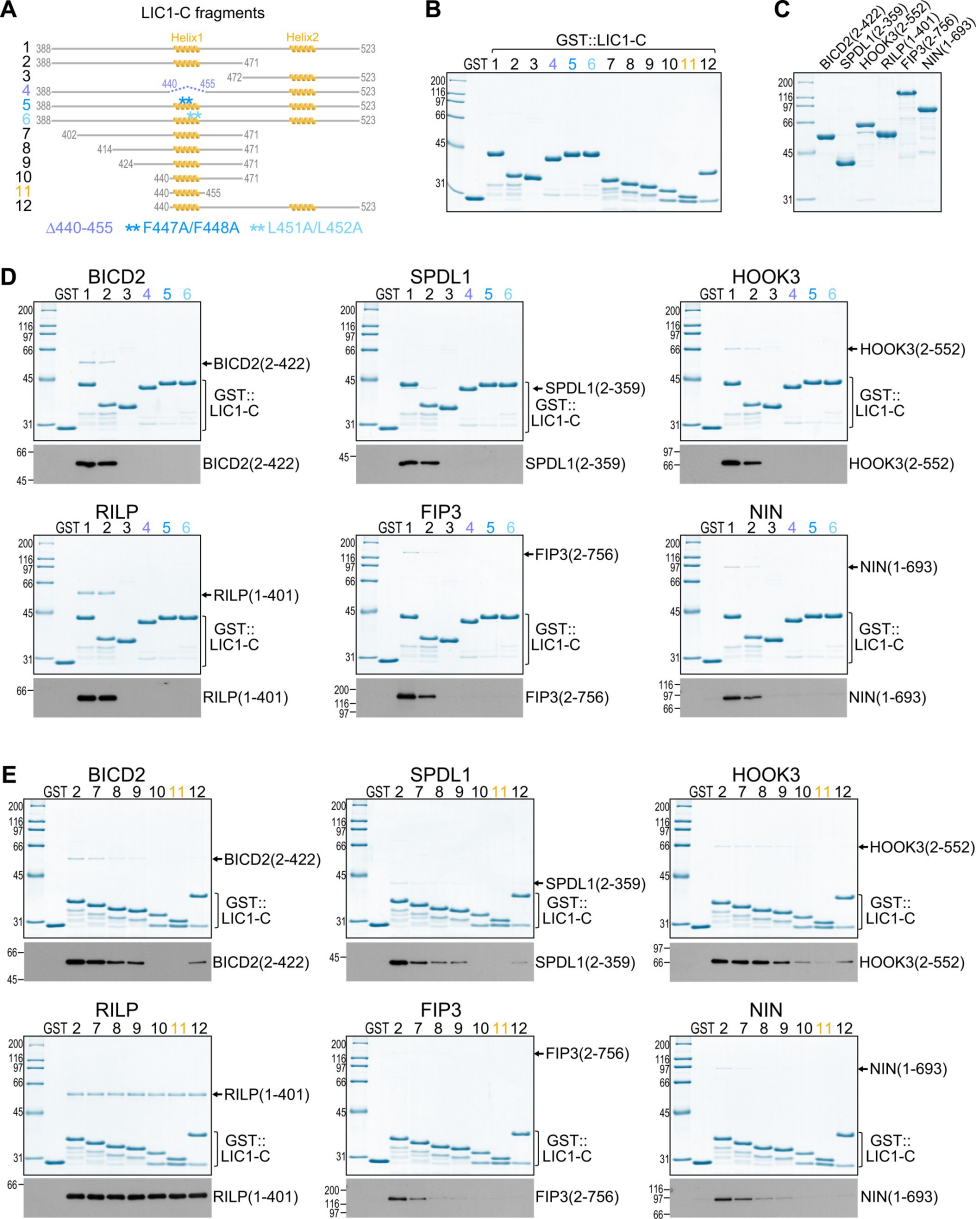
LIC1 C-terminal helix 1, but not helix 2, is essential for binding to structurally diverse adaptors. (A) LIC1-C fragments used for binding assays. (B, C) Coomassie Blue–stained protein gels of purified GST::LIC1-C fragments (B) and purified N-terminal or full-length versions of adaptors (C) (also see S1B Fig). All adaptors contain a C-terminal Strep-tag II. (D, E) GST pull-downs using the proteins shown in (B) and (C). Protein fractions bound to beads were visualized on gels by Coomassie Blue staining and by immunoblot against the Strep-tag II. Molecular mass is indicated in kDa on the left. Each pull-down was performed at least three times. BICD2, bicaudal D homolog 2; FIP3, RAB11 family-interacting protein 3; GST, glutathione S-transferase; HOOK3, Hook homolog 3; LIC1-C, C-terminal light intermediate chain 1; NIN, ninein; ppm, parts per million; RILP, RAB-interacting lysosomal protein; SPDL1, Spindly.

**Fig 4:**
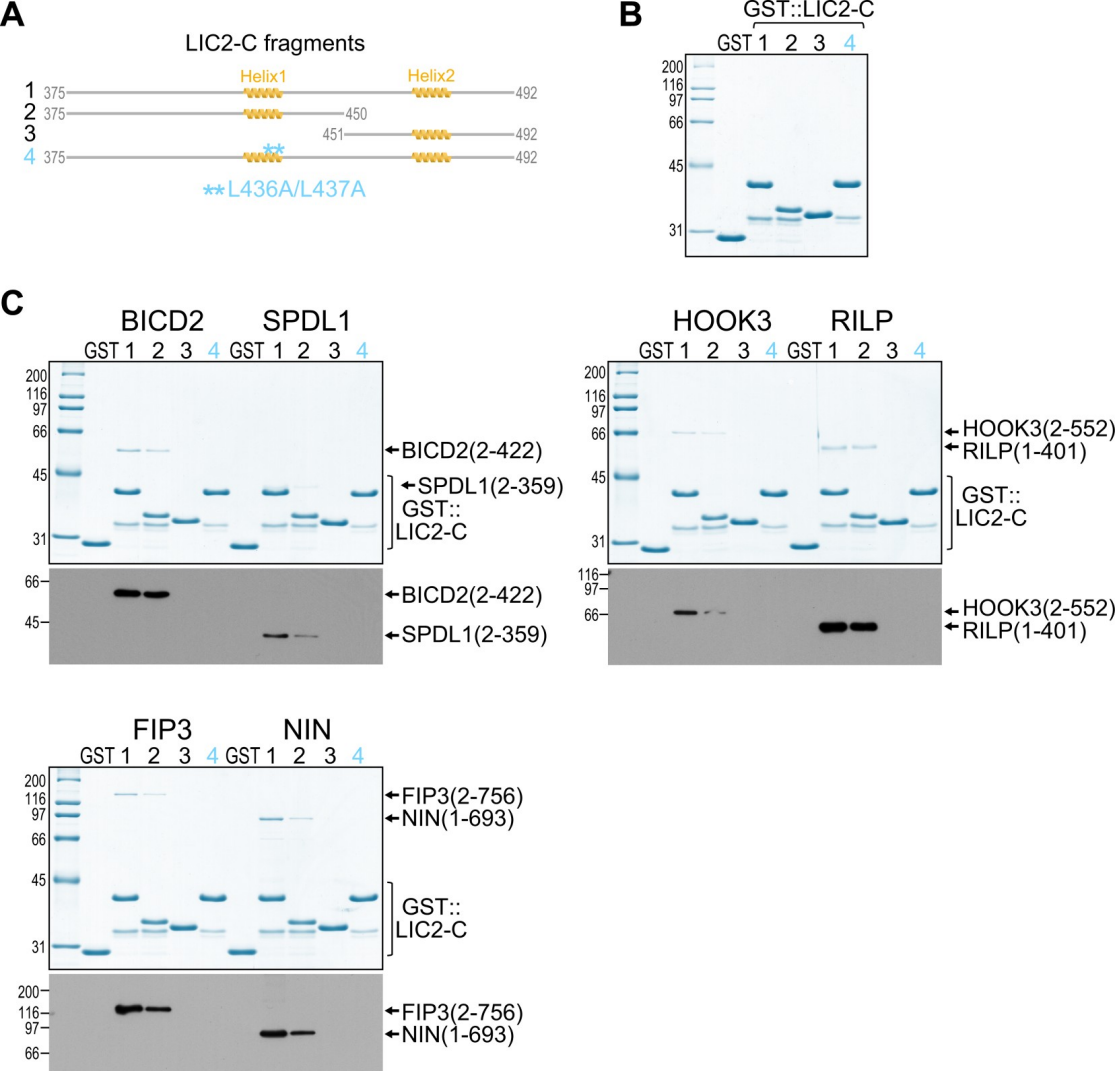
LIC2 also uses its C-terminal helix 1 for binding to adaptors. (A) LIC2-C fragments used for binding assays. (B) Coomassie Blue–stained protein gel of purified GST::LIC2-C fragments. (C) GST pull-downs using LIC2-C fragments shown in (B) and the Strep II–tagged adaptors shown in Fig 3C. Protein fractions bound to beads were visualized on gels by Coomassie Blue staining and by immunoblot against the Strep-tag II. Molecular mass is indicated in kDa on the left. Each pull-down was performed at least three times. BICD2, bicaudal D homolog 2; FIP3, RAB11 family-interacting protein 3; GST, glutathione S-transferase; HOOK3, Hook homolog 3; LIC2-C, C-terminal light intermediate chain 2; NIN, ninein; ppm, parts per million; RILP, RAB-interacting lysosomal protein; SPDL1, Spindly.

NMR spectroscopy and SPR analysis strongly suggested that helix 1 in LIC1-C is the major binding site for BICD2, SPDL1, and HOOK3. To directly test whether helix 1 is important for adaptor binding, we performed in vitro pull-down experiments using purified glutathione S-transferase (GST)-tagged versions of LIC1-C (Fig 3A and 3B) and LIC2-C (Fig 4A and 4B) as bait. In addition to BICD2(2–422), SPDL1(2–359), and HOOK3(2–552), we purified full-length versions of RILP (residues 1–401) and FIP3 (residues 2–756), as well as an N-terminal fragment of NIN (residues 1–693) (Fig 3C, S1B Fig). Using Coomassie Blue staining and immunoblotting for the Strep-tag II at the adaptor C terminus, all adaptors were readily detected in GST pull-downs with LIC1(388–523) and LIC1(388–471) (Fig 3D). By contrast, no significant binding was observed with LIC1(472–523). The same result was obtained with corresponding constructs of LIC2 (residues 375–492, 375–450, and 451–492) (Fig 4C). Deleting helix 1 in LIC1-C (Δ440–455), or mutating either the two phenylalanines or the two leucines in helix 1 to alanine (F447A/F448A and L451A/L452A) abrogated binding between LIC1-C and all six adaptors (Fig 3D). Mutating the two leucines in helix 1 of LIC2-C to alanine (L436A/L437A) had the same effect (Fig 4C). We conclude that helix 1 in LIC1-C and LIC2-C is essential for binding to cargo adaptors that are diverse in structure and function.

### Helix 1 is sufficient for binding to RILP, but other adaptors require additional contacts with LIC1-C

Next, we asked whether helix 1 of LIC1-C (residues 440–455) was sufficient to bind adaptors. In GST pull-down experiments, RILP bound robustly to LIC1(388–523) and LIC1(440–455) (Fig 3A, 3B and 3E). SPR experiments in which LIC1-C was immobilized through its N-terminal GST tag confirmed that RILP binds LIC1(440–455) with similar affinity as LIC1(388–523) (*K*_*D*_ of 1.67 ± 0.21 and 1.18 ± 0.14 μM, respectively; S6 Fig). We conclude that helix 1 of LIC1-C is sufficient for RILP binding. By contrast, none of the other five adaptors were detected in GST pull-downs with LIC1-C helix 1, suggesting that efficient binding requires additional segments in LIC1-C (Fig 3E).

NMR spectroscopy suggested that, in addition to helix 1, residues N-terminal to helix 1, as well as residues in helix 2, also make contact with adaptors. To investigate the contribution of these sites to adaptor binding, we first truncated LIC1(388–471) from its N terminus to generate progressively smaller fragments (residues 402–471, 414–471, 424–471, and 440–471), all of which retained helix 1 (Fig 3A). SPDL1(2–359), BICD2(2–422), HOOK3(2–552), FIP3(2–756), and NIN(1–693) showed reduced binding to LIC1-C with decreasing fragment size, whereas RILP bound equally well to all LIC1-C fragments (Fig 3E). Thus, for most adaptors, efficient binding to LIC1-C requires residues located N-terminally of helix 1. To assess the contribution of helix 2, we generated a fragment from the beginning of helix 1 until the end of LIC1-C (residues 440–523). Although SPDL1(2–359), BICD2(2–422), and HOOK3(2–552) were not detected in pull-downs using LIC1(440–455), they bound weakly to LIC1(440–523), potentially reflecting a modest contribution of helix 2 to the interaction (Fig 3E). Overall, our binding experiments with purified components demonstrate that helix 1 of LIC-C is essential for the interaction with adaptors but suggest that most adaptors also need to make additional contacts with the flexible LIC-C scaffold for efficient binding. Specifically, residues preceding helix 1 appear to make a significant contribution. The exception is RILP, for which LIC-C helix 1 on its own is sufficient for binding.

### Point mutations in the C-terminal helix 1 of *C*. *elegans* LIC cause severe developmental defects, impair locomotion, and shorten life span

**Fig 5:**
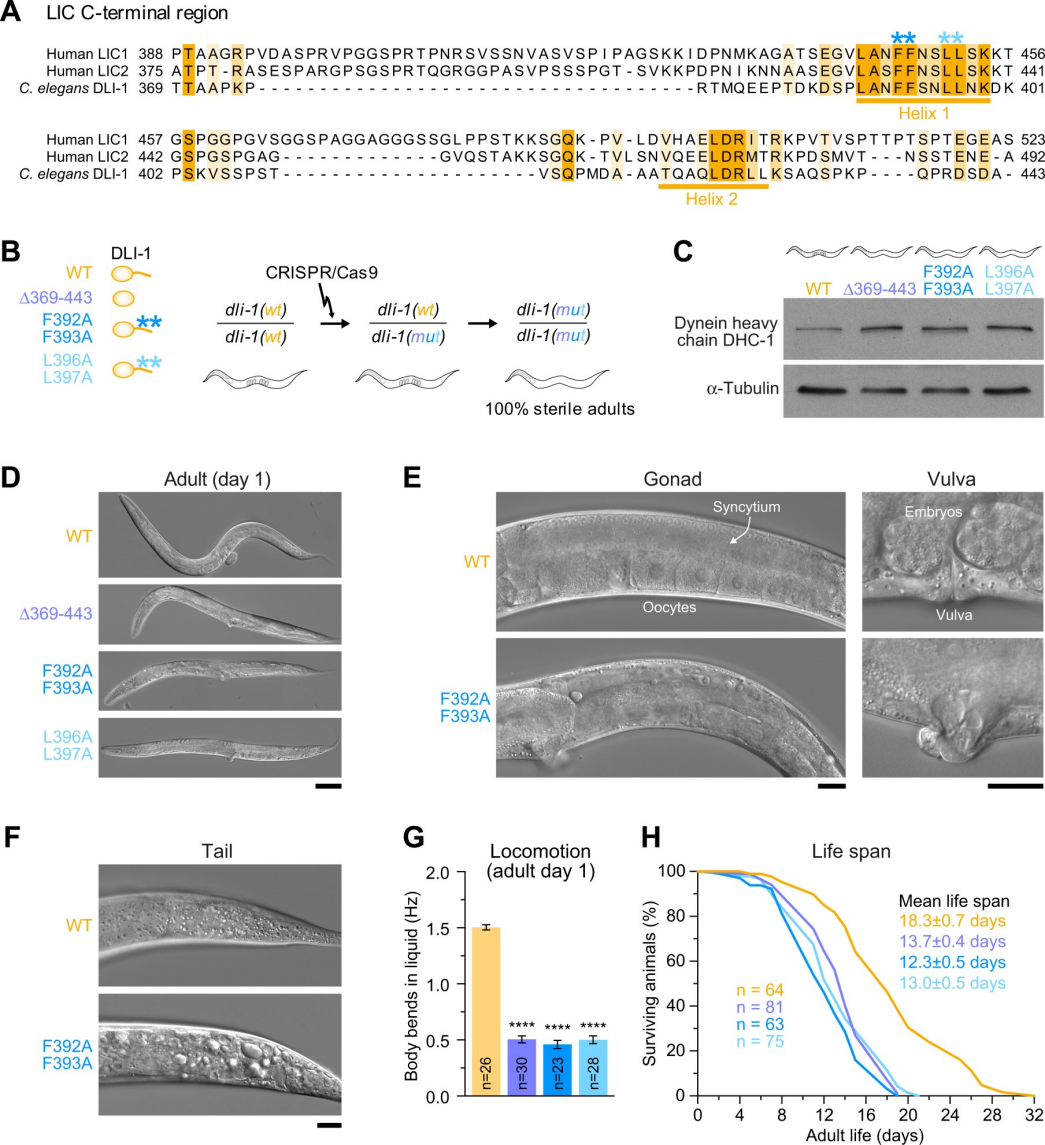
Point mutations in the C-terminal helix 1 of *C*. *elegans* DLI-1 cause severe developmental defects, impair locomotion, and shorten life span. (A) C-terminal sequence alignment of the two human LIC paralogs and the sole LIC ortholog in *C*. *elegans*, DLI-1, showing high conservation of helix 1. Residues mutated to alanine are marked by asterisks. (B) (left) Cartoon showing DLI-1 WT and mutant versions (Δ369–443, F392A/F393A, or L396A/L397A) expressed in animals after CRISPR/Cas9-mediated genome editing. (right) Genetics of *dli-1* mutant alleles. Mothers heterozygous for the mutant *dli-1* allele contain a balancer chromosome with a WT copy, *dli-1(wt)*. Progeny homozygous for the mutant *dli-1* allele develop to adulthood but are sterile. All three *dli-1* mutants behave the same. (C) Immunoblot showing levels of DHC-1 in adults of *dli-1* mutants. α-Tubulin is used as a loading control. Also see S7 Fig. (D–F) Differential interference contrast images of animals at the day 1 adult stage, showing extensive morphological defects in *dli-1* mutants. Scale bars: (D) 100 μm; (E) and (F) 20 μm. (G) Locomotion of day 1 adult animals, assessed by determining body bending frequency in liquid medium. Graph shows mean ± SEM for *n* number of animals from two independent experiments. Statistical significance (mutant versus WT *dli-1*) was determined by one-way ANOVA on ranks (Kruskal-Wallis nonparametric test) followed by Dunn’s multiple comparison test. ****P < 0.0001. (H) Life span curves. Animals were collected as L4 larvae (day 0) and observed every 1–3 d until they died. Mean life span (± SEM) is indicated for *n* number of animals. Underlying data for Fig 5 can be found in S1 Data. CRISPR/Cas9, clustered regularly interspaced short palindromic repeat/CRISPR-associated 9; DHC-1, dynein heavy chain 1; DLI-1, dynein LIC 1; LIC, light intermediate chain; WT, wild type.

To determine whether helix 1 of LIC-C contributes to dynein function in vivo, we turned to the animal model *C*. *elegans*, which expresses a single dynein LIC, *dli-1* [30]. Just as in other LIC orthologs, the C-terminal helix 1 is highly conserved in DLI-1 (Fig 5A). We used clustered regularly interspaced short palindromic repeat/CRISPR-associated 9 (CRISPR/Cas9)–mediated genome editing to mutate either the two phenylalanines or the two leucines in helix 1 to alanine (F392A/F393A and L396A/L397A), in analogy to the human LIC-C mutations we characterized in vitro (Fig 5B). For comparison, we also deleted the entire DLI-1 C-terminal region (Δ369–443). For all three *dli-1* mutants, homozygous offspring from heterozygous mothers developed to adulthood but were sterile, suggesting that the C-terminal region of DLI-1, and helix 1 in particular, is essential for dynein function in vivo (Fig 5B). Because RNA interference (RNAi)-mediated depletion of DLI-1 showed that DLI-1 is required for the stability of dynein HC 1 (DHC-1; S7 Fig), we addressed the possibility that the phenotype of our *dli-1* mutants was a consequence of reduced DHC-1 levels. Immunoblotting of homozygous adults with an antibody against DHC-1 demonstrated that this was not the case. On the contrary, DHC-1 levels appeared slightly increased in all three *dli-1* mutants (Fig 5C and S7B Fig). This indicates that the C-terminal DLI-1 mutants do not interfere with the binding of the N-terminal GTPase-like domain to DHC-1.

Differential interference contrast imaging revealed severe morphological defects in day 1 adults homozygous for the *dli-1* mutations (Fig 5D, 5E and 5F). All three *dli-1* mutants had a slightly dumpy body with a protruding vulva and a highly disorganized and underdeveloped gonad (Fig 5D and 5E), which is indicative of problems with postembryonic cell divisions. Furthermore, the prevalence of vacuoles suggested widespread necrotic-like cell death (Fig 5F). Consistent with this, *dli-1* mutants exhibited severe defects in locomotion (Fig 5G), which was assessed by determining the body bending frequency in liquid medium (wild type, 1.50 ± 0.02 Hz; Δ369–443, 0.50 ± 0.03 Hz; F392A/F393A, 0.46 ± 0.04 Hz; L396A/L397A, 0.50 ± 0.03 Hz). *Dli-1* mutants had a significantly shorter life span compared to wild-type animals (wild type, 18.3 ± 0.7 d; Δ369–443, 13.7 ± 0.4 d; F392A/F393A, 12.3 ± 0.5 d; L396A/L397A, 13.0 ± 0.5 d) (Fig 5H). None of the *dli-1* mutant animals lived beyond 24 d, whereas 27% of wild-type animals did. We conclude that deletion of the DLI-1 C-terminal disordered region and point mutations in the conserved helix 1 cause similar defects in development, locomotion, and life span. These phenotypes are reminiscent of those reported for other dynein mutants, including null mutants of *dli-1* [30,31].

### Point mutations in the C-terminal helix 1 of DLI-1 cause accumulation of membrane cargo at nerve endings of mechanosensory neurons and perturb retrograde axonal transport

**Fig 6:**
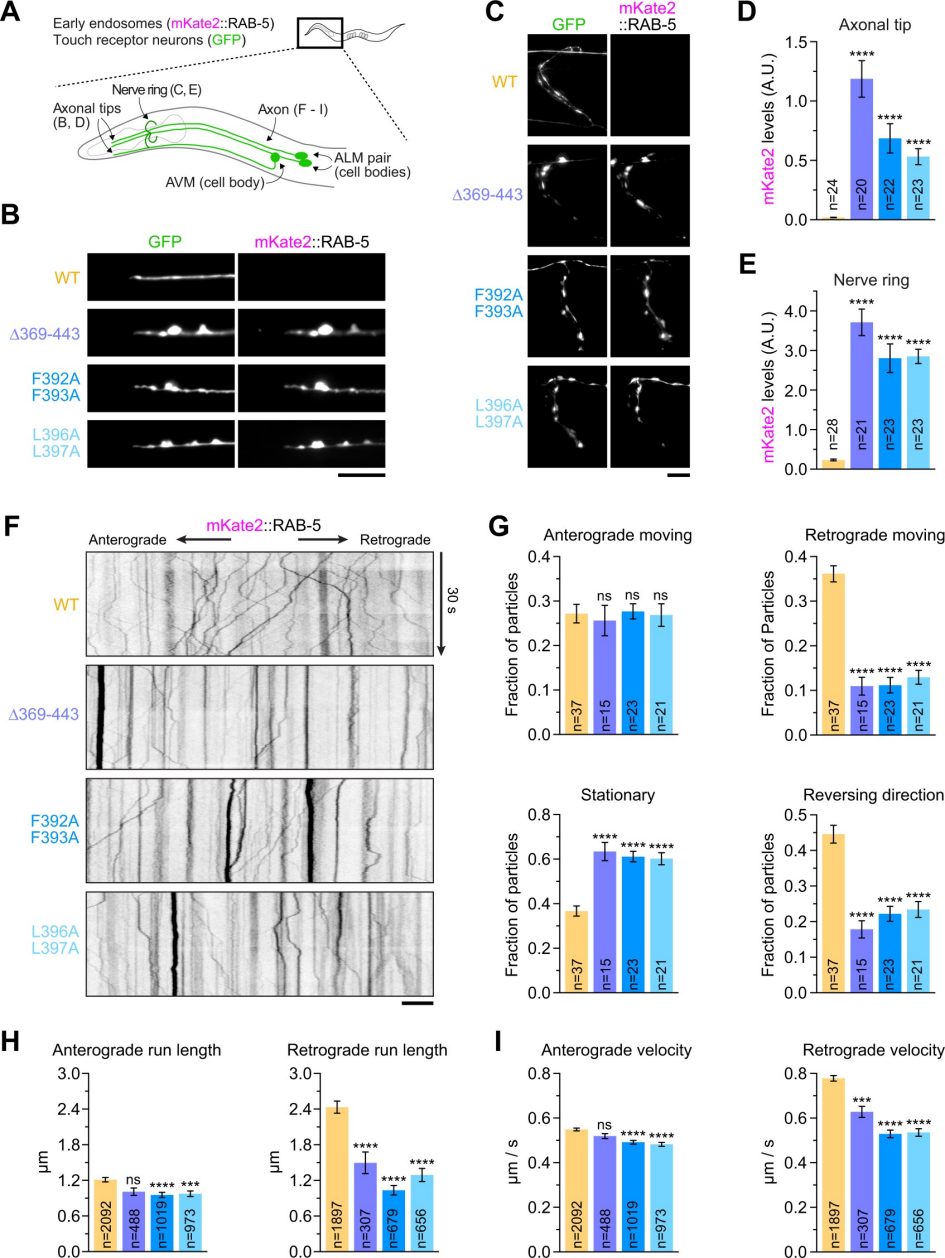
C-terminal *dli-1* mutations disrupt retrograde axonal transport of early endosomes. (A) Cartoon showing the architecture of anterior touch receptor neurons. Animals coexpress mKate2::RAB-5, a marker for early endosomes, and soluble GFP in touch receptor neurons. ALM and AVM are neurons that extend processes into the nose and the nerve ring. Results shown in (B–I) derive from imaging specific regions in these neurons, as indicated. (B, C) Fluorescence images of axonal tips (B) and nerve rings (C) in day 1 adults, showing misaccumulation of early endosomes in *dli-1* mutants. Scale bars, 10 μm. (D, E) Quantification of early endosome misaccumulation in axonal tips (D) and nerve rings (E), using fluorescence intensity measurements of mKate2::RAB-5 in images as shown in (B) and (C), respectively. Graphs represent the mean ± SEM signal in A.U. for *n* number of neurons imaged in two independent experiments. Statistical significance (mutant versus WT *dli-1*) was determined by one-way ANOVA on ranks (Kruskal-Wallis nonparametric test) followed by Dunn’s multiple comparison test. ****P < 0.0001. Also see S1 Movie. (F) Kymographs of mKate2::RAB-5-labeled early endosome motility in axons of ALM neurons, imaged at the L4 larval stage. Time-lapse sequences of confocal images were recorded every 200 ms in an axonal region approximately 50 μm away from the cell body, which is positioned to the right. Scale bar, 10 μm. (G–I) Quantification of early endosome motility, based on the analysis of kymographs as shown in (F). Graphs represent the mean ± SEM. For (G), *n* is the total number of axons. For (H) and (I), *n* is the total number of segments within tracks of moving particles framed by a pause or a reversal. Results are derived from 2–5 independent imaging sessions. Statistical significance was determined as described for (D, E). ****P < 0.0001; ***P < 0.001; ns indicates *P* > 0.05. Also see S8A Fig. Underlying data for Fig 6 can be found in S1 Data. ALM, anterior lateral mechanosensory; A.U., arbitrary units; AVM, anterior ventral mechanosensory; GFP, green fluorescent protein; ns, not significant; WT, wild type.

Next, we sought to assess the impact of the C-terminal *dli-1* mutations on dynein-dependent cargo transport. We chose to examine the distribution and transport kinetics of membrane cargo in axons of touch receptor neurons, which are mechanosensory neurons whose processes extend along the length of the animal just underneath its cuticle (Fig 6A). Axonal microtubules in touch receptor neurons are polarized with their plus ends oriented toward the axonal tip, and dynein is therefore responsible for retrograde transport of cargo toward the cell body [31,32]. Using Mos transposase-mediated single-copy insertion [33], we constructed transgenic animals expressing mKate2::RAB-5 from the *mec-7* promoter and crossed them with animals expressing soluble green fluorescent protein (GFP) from the *mec-4* promoter (allele *zdIs5*). This allowed simultaneous visualization of early endosomes (mKate2), a cargo of dynein, and neuronal morphology (GFP) (Fig 6A). In contrast to control day 1 adults, all three *dli-1* mutants exhibited a pronounced misaccumulation of mKate2::RAB-5 at the axonal tips and nerve ring synapses of anterior lateral mechanosensory (ALM) and anterior ventral mechanosensory (AVM) neurons (Fig 6B, 6C, 6D and 6E). This defect resembled the previously described misaccumulation of synaptic vesicle proteins in touch receptor neurons of dynein mutants [31].

To determine how *dli-1* mutants affect the kinetics of axonal transport, we imaged mKate2::RAB-5-labeled early endosomes at high temporal resolution in an axonal segment close to the cell body of ALM neurons at the larval L4 stage (Fig 6A and 6F and S1 Movie). Quantitative analysis of kymographs revealed that the three *dli-1* mutants had near-identical defects in axonal transport. The fraction of particles moving in the retrograde direction was reduced significantly, with a corresponding increase in the fraction of stationary particles (Fig 6G). Analysis of individual runs showed that particles spent less time in retrograde motion and paused more frequently and for longer periods of time (S8A Fig). Particles also had a decreased run length and moved at reduced speed, especially in the retrograde direction (Fig 6H and 6I). Overall, this analysis revealed that all three *dli-1* mutants strongly impaired retrograde axonal transport of early endosomes, consistent with compromised dynein function.

We also examined the axonal distribution of two additional types of dynein cargo, each labeled with a marker expressed from a single-copy transgene: synaptic vesicles (SNB-1 [synaptobrevin 1]::mKate2) and mitochondria (TOMM-20 [translocase of outer mitochondrial membrane 20][1–54]::mKate2). Because the three *dli-1* mutants had identical defects in the distribution and transport kinetics of mKate2::RAB-5, we restricted our analysis to the *dli-1(L396A/L397A)* mutant. We found that *dli-1(L396A/L397A)* animals misaccumulated SNB-1::mKate2 at axonal tips (S8B, S8C and S8D Fig), similar to the results of a prior study that examined the distribution of SNB-1::GFP in the null mutant *dli-1(js351)* [31].

**Fig 7:**
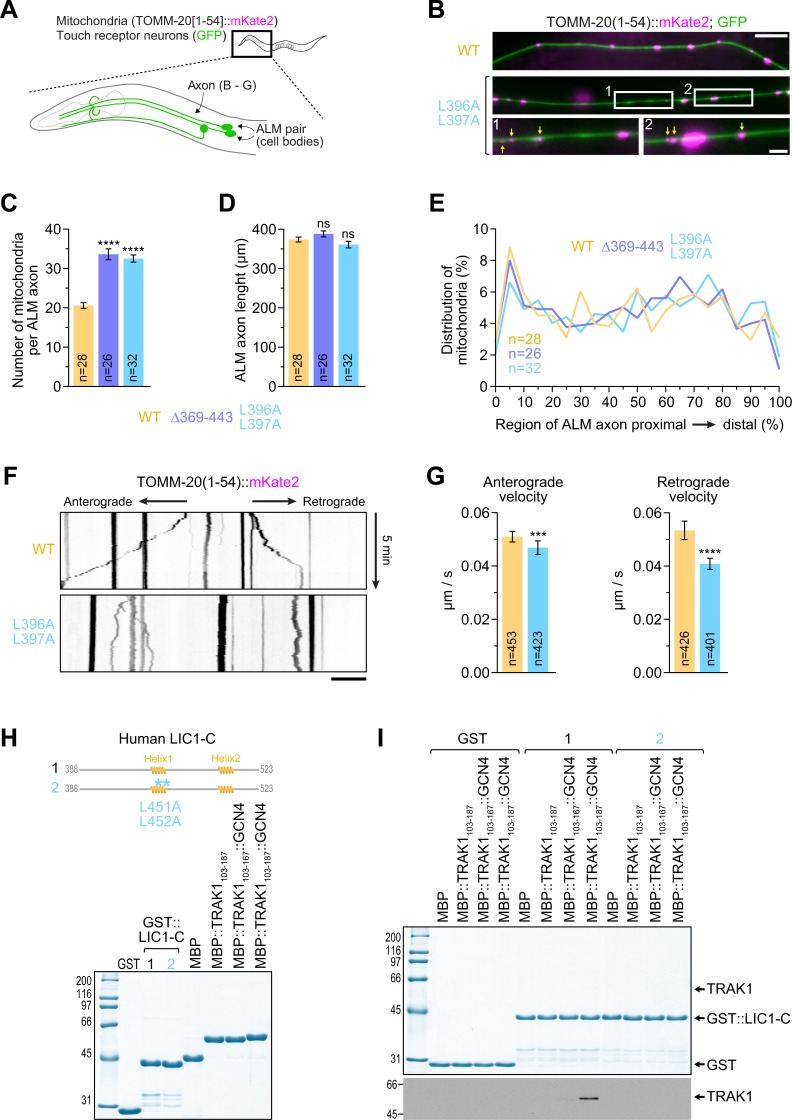
C-terminal *dli-1* mutants have increased numbers of mitochondria that move with reduced retrograde velocity. (A) Cartoon highlighting the axons of ALM neurons examined in (B–G). Animals coexpress TOMM-20(1–54)::mKate2, a marker for mitochondria, and soluble GFP in these neurons. (B) Fluorescence images of ALM axons in animals at the day 1 adult stage. Blow-ups highlight the prevalence of small (<1 μm) TOMM-20(1–54)::mKate2 puncta in the *dli-1(L396A/L397A)* mutant. Scale bars, 10 μm; blow-ups, 2.5 μm. (C, D) Total number of mitochondria per ALM axon (C) and axonal length (D) in day 1 adults. Graphs represent the mean ± SEM. Statistical significance (mutant versus WT *dli-1*) was determined by one-way ANOVA on ranks (Kruskal-Wallis nonparametric test) followed by Dunn’s multiple comparison test. ****P < 0.0001; ns indicates *P* > 0.05. (E) Relative distribution of mitochondria along the ALM axon from the proximal (0%) to the distal (100%) end in day 1 adults. Graph corresponds to the mean, and the number *n* of axons imaged for each condition is indicated. (F) Kymographs of mitochondrial motility in axons of ALM neurons, imaged at the L4 larval stage. Time-lapse sequences of confocal images were recorded every 5 s in an axonal region approximately 50 μm away from the cell body, which is positioned to the right. Scale bar, 10 μm. Also see S2 Movie. (G) Quantification of mitochondrial transport velocity, based on the analysis of kymographs as shown in (F). Graphs represent the mean ± SEM. *n* is the total number of segments within tracks of moving particles framed by a pause or a reversal. Results are derived from 5–8 independent imaging sessions. Statistical significance was determined as described for (C, D). ****P < 0.0001; ***P < 0.001. Also see S8E and S8F Fig. (H) Coomassie Blue–stained protein gel of purified GST-tagged human LIC1-C and purified MBP::TRAK1 fragments containing a C-terminal Strep-tag II (also see S1B Fig). Two different versions of the first coiled-coil segment of TRAK1 were fused to a fragment of the yeast transcriptional activator GCN4 for artificial dimerization. (I) GST pull-downs using the proteins shown in (H). Protein fractions bound to beads were visualized on gels by Coomassie Blue staining and by immunoblot against the Strep-tag II. Molecular mass is indicated in kDa on the left. Each pull-down was performed at least three times. Underlying data for Fig 7 can be found in S1 Data. ALM, anterior lateral mechanosensory; GCN4, general control nondepressible 4; GFP, green fluorescent protein; GST, glutathione S-transferase; LIC1-C, C-terminal light intermediate chain 1; MBP, maltose-binding protein; ns, not significant; TRAK1, trafficking kinesin-binding protein 1; WT, wild type.

When examining the distribution of TOMM-20(1–54)::mKate2 particles (Fig 7A), we found that axons of ALM neurons contained an average of 21 ± 1 mitochondria in control day 1 adults, corresponding to a density of 5.6 mitochondria per 100 μm, and that mitochondria were evenly distributed along axons (Fig 7B, 7C, 7D and 7E). These findings are consistent with prior reports [34,35]. Mitochondria remained evenly distributed in ALM axons in *dli-1(L396A/L397A)* animals (Fig 7E). However, the average number of axonal mitochondria increased to 33 ± 1, corresponding to a density of 9 mitochondria per 100 μm (Fig 7C and 7D). Close inspection of mitochondrial morphology suggested that the increase was primarily due to an excess of small TOMM-20(1–54)::mKate2 puncta, which may represent fragmented mitochondria (Fig 7B). Thus, the *dli-1(L396A/L397A)* mutation does not result in distribution bias of mitochondria along the axon but appears to promote mitochondrial fission. Similar results were obtained for the *dli-1(Δ369–443)* mutation (Fig 7C, 7D and 7E). Analysis of mitochondrial transport kinetics in *dli-1(L396A/L397A)* animals revealed a reduction in the speed of transport, with a more pronounced effect in the retrograde direction (Fig 7F and 7G, and S2 Movie). However, none of the other transport parameters were significantly altered (S8E and S8F Fig).

Given the modest impact of the *dli-1(L396A/L397A)* mutation on mitochondrial transport, we asked whether the mitochondrial adaptor TRAK1, which contains a CC1 box similar to SPDL1 and BICD2, binds LIC1. GST pull-downs with purified human proteins showed that maltose-binding protein (MBP)::TRAK1(103–187), which corresponds to the first coiled-coil segment, was able to bind GST::LIC1(388–523), albeit weakly and only when artificially dimerized using a general control nondepressible 4 (GCN4) sequence (Fig 7H and 7I). The interaction was abolished when the two leucines in LIC1-C helix 1 were mutated, suggesting that LIC1 binds TRAK1 through a similar mechanism as the other adaptors.

### The C-terminal helix 2 of DLI-1 is largely dispensable for dynein function in vivo

**Fig 8:**
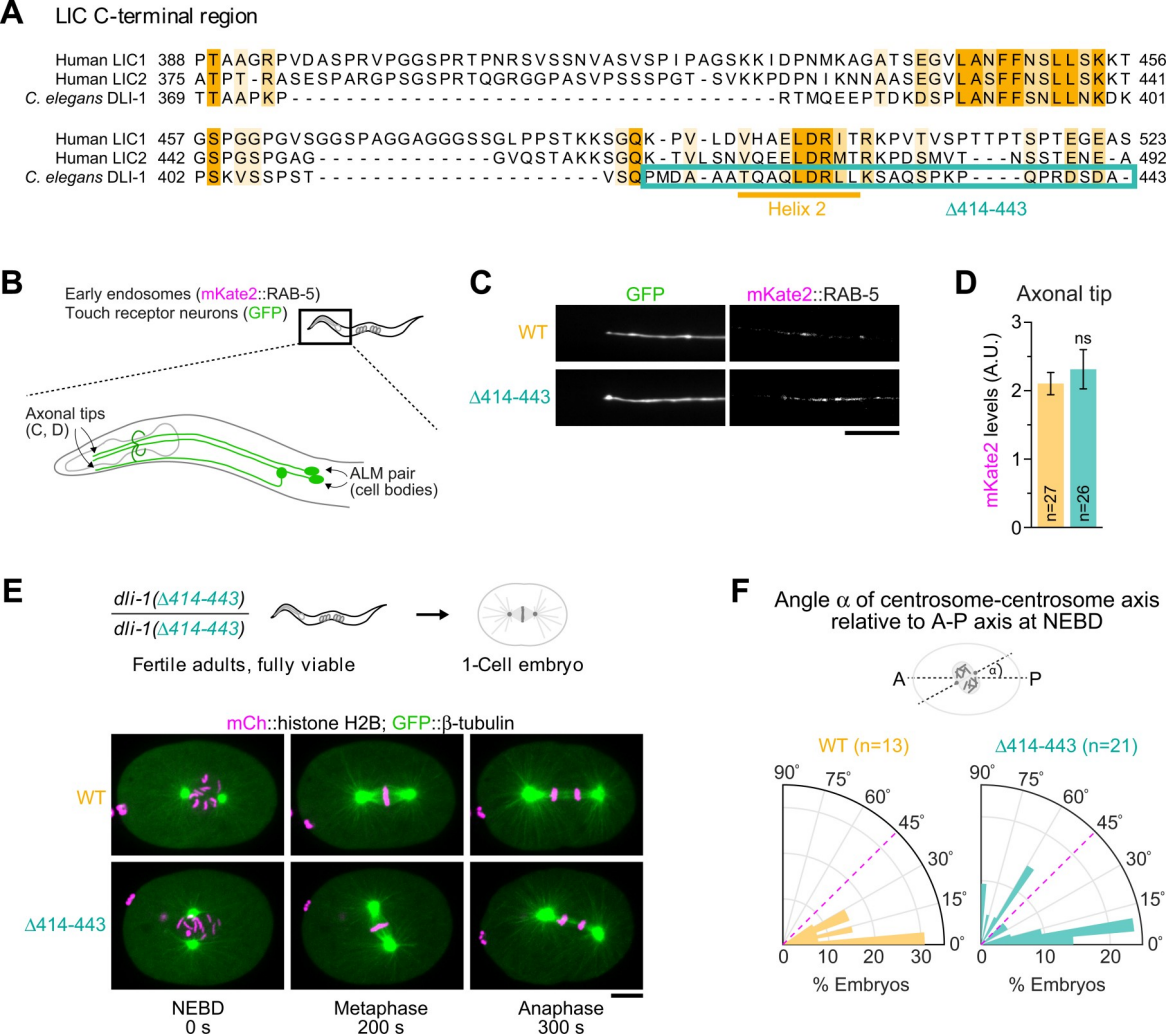
Deletion of the C-terminal helix 2 in DLI-1 affects mitotic spindle positioning in the one-cell embryo. (A) Sequence alignment of human and *C*. *elegans* LIC-C showing the deleted region in DLI-1 that includes the conserved helix 2. (B) Cartoon highlighting the axons of ALM neurons examined in (C) and (D). Animals coexpress mKate2::RAB-5 and soluble GFP in these neurons. (C) Fluorescence images of ALM axonal tips in day 1 adults. Scale bar, 10 μm. (D) Quantification of mKate2::RAB-5 levels at axonal tips, using fluorescence intensity measurements in images as shown in (C). Graph represents the mean ± SEM signal in A.U. for *n* number of neurons imaged in two independent experiments. Statistical significance (mutant versus WT *dli-1*) was determined with the Mann-Whitney test. ns indicates *P* > 0.05. (E) (top) Cartoon illustrating that animals homozygous for *dli-1(*Δ*414–443)* are viable and fertile. (bottom) Stills from time-lapse sequences in the one-cell embryo showing the delay in mitotic spindle orientation in *dli-1(*Δ*414–443)*. Scale bar, 10 μm. (F) (top) Cartoon showing the angle (α) between the C-C axis and the A-P axis in one-cell embryos at NEBD. (bottom) Rose diagrams of angle α measured at NEBD in images as shown in (E). A significant fraction of *dli-1(*Δ*414–443)* embryos have a severely tilted C-C axis relative to controls (α > 45°; Fisher’s exact test, *P* = 0.029). Underlying data for Fig 8 can be found in S1 Data. ALM, anterior lateral mechanosensory; A-P, anterior–posterior; A.U., arbitrary units; C-C, centrosome–centrosome; DLI-1, dynein LIC 1; GFP, green fluorescent protein; LIC, light intermediate chain; LIC-C, C-terminal LIC; NEBD, nuclear envelope breakdown; ns, not significant; WT, wild type.

Just like helix 1, helix 2 in LIC-C shows high sequence conservation, including in *C*. *elegans* (residues 420–428; Fig 8A and S1A Fig). To assess the role of helix 2 in vivo, we used genome editing to generate animals that express DLI-1 without its C-terminal 30 residues (Δ414–443). This truncated version of DLI-1 retains helix 1 (residues 388–400) (Fig 8A). In contrast to the helix 1 point mutants, homozygous *dli-1(*Δ*414–443)* animals were fully viable and fertile (embryonic viability 98.4% ± 0.6% and 97.3% ± 0.7% for wild type [>300 progeny, 12 mothers] and mutant [>300 progeny, 14 mothers], respectively). Furthermore, we observed no increase in mKate2::RAB-5 signal at axonal tips of touch receptor neurons, indicating that dynein-dependent transport of early endosomes was not significantly affected in this mutant (Fig 8B, 8C and 8D). Because homozygous *dli-1(*Δ*414–443)* animals produced progeny, we were able to examine the first mitotic division of embryos coexpressing GFP::β-tubulin and mCherry::histone H2B, which mark microtubules and chromosomes, respectively (Fig 8E). During mitosis, dynein-dependent pulling on centrosome-nucleated microtubules is essential for the separation and positioning of centrosomes, which form the spindle poles after nuclear envelope breakdown (NEBD). At the time of NEBD in control animals, centrosomes were fully separated and the centrosome–centrosome axis was oriented approximately parallel to the anterior–posterior axis (Fig 8E and 8F). In *dli-1(*Δ*414–443)* embryos, centrosomes separated normally, but the centrosome–centrosome axis was frequently severely tilted relative to the anterior–posterior axis (angle > 45° in 7 out of 21 mutant embryos versus 0 out of 13 controls), resulting in misoriented spindles. Despite initial misorientation, spindles ultimately correctly aligned with the anterior–posterior axis, and chromosome segregation completed without defects. The delay in spindle orientation indicates a subtle impairment of dynein-dependent pulling forces [36,37]. To further probe the functional significance of helix 2, we combined the *dli-1(*Δ*414–443)* mutation with a null allele of the dynein cofactor *nud-2*, *nud-2(ok949)*, which represents a sensitized dynein background that also exhibits a spindle orientation delay and that we had previously exploited for an enhancer screen [36,38]. We observed neither an increase in lethality nor enhanced defects in spindle orientation in the double mutant relative to the single mutants (embryonic viability 85.9% ± 1.6% and 89.8% ± 1.4% for *ok949* [>300 progeny, 15 mothers] and *ok949; dli-1[*Δ*414–443]* [>300 progeny, 19 mothers], respectively), consistent with the idea that dynein function in *dli-1(*Δ*414–443)* animals is relatively unperturbed.

We conclude that helix 2 of the DLI-1 C-terminal region makes a modest contribution to dynein-dependent pulling forces during mitosis but overall plays a minor role in vivo compared to helix 1, which is critical for dynein function. These results are consistent with our biochemical characterization of human LIC-C in vitro, which shows that helix 1, but not helix 2, is essential for the interaction between LIC-C and cargo adaptors.

## Discussion

How dynein achieves specificity for cargo is a key question underlying the functional versatility of the motor. Studies over the past few years have revealed that the N-terminal region of cargo adaptor proteins helps bring together the motor with its essential cofactor dynactin and that multiple distinct protein–protein interactions participate in the assembly of a processive dynein-dynactin-adaptor transport machine. Here, we dissected the structural determinants and the functional relevance of the interaction between the C-terminal region of the dynein LIC subunit and cargo adaptors. We present high-resolution molecular evidence that LIC-C is disordered and that a conserved segment with helical propensity, helix 1, is the main binding site for seven adaptors that differ in structure and cargo specificity. Our results agree with recent work that identified LIC1-C helix 1 as the critical structural element for the interaction with HOOK1/3, SPDL1, and BICD2 [13], and we extend these findings to RILP, NIN, FIP3, and TRAK1. The highly conserved phenylalanines and leucines of the amphipathic helix 1 are essential for the interaction, which occurs with single-digit micromolar affinity for SPDL1, BICD2, HOOK3, and RILP, suggesting a similar binding mechanism. NMR spectroscopy analysis shows that helix 1 is transient. The presence of transiently sampled secondary elements in intrinsically disordered proteins is common and may facilitate the transition to more rigid states upon binding [39–41]. The observed signal quenching upon binding to adaptors is unlikely to be caused entirely by an increased local tumbling time. Instead, micro-millisecond conformational exchange between various states of the helical elements or binding kinetics contribute significantly to the quenching. Both mechanisms suggest residual structural disorder. Indeed, various examples of disordered proteins that maintain partial disorder during interaction with other proteins have recently been reported [40,42–44]. A prior study used X-ray crystallography to show that LIC1-C helix 1 inserts into a hydrophobic cleft on HOOK3(1–160) [13]. An analogous hydrophobic cleft is likely formed by the CC1 box, the LIC1-C binding region in SPDL1 and BICD2 [13,16]. CC1 boxes are also present in other adaptors whose N-terminal regions are structurally similar to SPDL1 and BICD2, such as HAP1 and TRAK [45]. Consistent with this, we show that the first coiled-coil segment of TRAK1 interacts with LIC1-C in a helix 1-dependent manner. RILP, NIN, and FIP3 contain neither a Hook domain nor a CC1 box. The structural element that accommodates the LIC-C helix 1 in these adaptors remains to be identified.

LIC1-C helix 1 is essential for adaptor binding, but we find that helix 1 on its own—i.e., LIC1(440–455)—is not sufficient for efficient binding to 5 out of 6 adaptors tested, RILP being the exception. Pull-down assays with LIC1-C truncations show that segments N-terminal to helix 1 contribute to adaptor binding. LIC1-C binding to SPDL1, FIP3, and NIN appears to be particularly sensitive to N-terminal LIC1-C truncations, as we find that LIC1(424–471) has markedly reduced affinity relative to LIC1(388–471). This is in agreement with NMR analysis, which indicates that residues 418–421 participate in the interaction with SPDL1. NMR analysis also indicates that LIC1-C helix 2 participates in the interaction with adaptors, but in vitro binding experiments show that the contribution of helix 2 to the overall binding affinity is relatively minor.

Vertebrates possess two LIC paralogs that differ significantly in their C-terminal region. Prior studies in cultured cells suggest that there is functional redundancy among LIC1 and LIC2 but also hint at functional specialization in dynein-dependent cell division processes and membrane trafficking [46–53]. One attractive possibility is that the C-terminal regions of LIC1 and LIC2 discriminate between adaptors. Our results show that LIC1 and LIC2 use the same helix 1–based mechanism for adaptor binding, and we did not detect any striking differences in the ability of the two C-terminal regions to interact with our set of six adaptors. Nevertheless, it is plausible that LIC1 and LIC2 exhibit subtle preferences for specific adaptors. For example, we find that NIN binds slightly better to LIC2 than LIC1. Posttranslational modifications could possibly further modulate the affinity for adaptors in an isoform-specific manner.

Previous work in vitro showed that the dynein-dynactin-adaptor assembly fails to form in HOOK3 and SPDL1 mutants that cannot bind to LIC1 [16,17] and that addition of excess LIC1-C helix 1 inhibits processive movement mediated by HOOK3 or BICD2 fragments in motility assays [13]. Here, we present direct evidence that LIC-C helix 1 is important for dynein function in vivo. Like other invertebrates, the nematode *C*. *elegans* expresses only one LIC isoform, which facilitates the identification of functionally critical regions. In all assays, the outcome of mutating the two conserved phenylalanines or leucines in the C-terminal helix 1 of DLI-1 is identical to that of deleting the entire C-terminal region. Unlike DLI-1 depletions, the phenotype of the C-terminal DLI-1 mutations cannot be attributed to destabilization of DHC-1 and therefore likely reflects defective adaptor binding. On the animal level, sterility and other developmental defects suggest failure of postembryonic cell division, similar to what was described for *dli-1* null mutants [30,31]. We show on the cellular level that the C-terminal DLI-1 mutations disrupt the distribution of early endosomes and presynaptic vesicles in axons of touch receptor neurons. Since we find that axonal length is normal in day 1 adults of *dli-1(L396A/L397A)* and *dli-1(*Δ*369–443)* mutants, this is unlikely to be an indirect consequence of a developmental defect. Instead, the misaccumulation at the axonal tip is suggestive of impaired retrograde transport by dynein, as previously described for other dynein mutants, including the *dli-1* null mutant *js351* [31]. Consistent with this, we show that the frequency, velocity, and run length of early endosome movement is significantly decreased in our *dli-1* mutants, with a predominant effect on retrograde movement. Neurodegeneration (i.e., axon beading) starts to become evident in *dli-1* mutant day 1 adults and likely contributes to the animals' severe locomotory deficit and shortened life span.

The residual retrograde movement of early endosomes in axons of *dli-1* mutants could indicate that dynein retains a limited ability to form processive dynein-dynactin-adaptor complexes without the interaction between DLI-1 and adaptors. This may also explain the modest effect of *dli-1* mutants on mitochondrial transport. However, it is difficult to rule out that a small fraction of wild-type maternal DLI-1, passed on from the heterozygous mother to homozygous mutant progeny, could persist in touch receptor neurons at the L4 stage and promote residual dynein activity. Nevertheless, the observation that *dli-1* mutants have a more pronounced effect on early endosomes and synaptic vesicles than on mitochondria indicates that DLI-1 binding to adaptors may not be equally important for all cargo transport.

In contrast to the point mutants in the DLI-1 C-terminal helix 1, there are no obvious developmental defects in animals expressing the DLI-1(Δ414–443) mutant that lacks the C-terminal helix 2, and mKate2::RAB-5 is not misaccumulated at the axonal tip of touch receptor neurons in this mutant. We have not examined neuronal cargo transport kinetics, but given that *dli-1(*Δ*414–443)* animals are healthy and propagate normally, any defects are unlikely to be substantial. We do, however, observe a delay in mitotic spindle positioning in one-cell *dli-1(*ΔΔ*414–443)* embryos. The mild phenotype contrasts with the failure of spindle assembly observed in one-cell embryos after DLI-1 depletion [30]. Furthermore, the mitotic defects of *dli-1(*Δ*414–443)* embryos are not enhanced by a null allele of the dynein cofactor *nud-2*, which partially compromises dynein [36,38]. Thus, analysis in vivo and in vitro indicates that LIC-C helix 2, despite its high sequence conservation, makes a relatively modest contribution to dynein function compared to helix 1.

Together with the work of Lee and colleagues [13], our study establishes the molecular mechanism used by LIC to interact with structurally diverse cargo adaptors. An interesting open question is whether LIC-C could have additional binding partners besides adaptors. Two recent cryo-EM studies revealed that two dyneins can be recruited by a single dynactin-adaptor complex [10,11]. In one of the structures, an extra density, most likely corresponding to LIC-C of the first dynein, packs against the N-terminal coiled-coil of BICDR1 while simultaneously contacting one of the HCs of the second dynein [10]. It is tempting to speculate that this interaction between LIC-C and HC facilitates the incorporation of a second dynein into dynein-dynactin-adaptor assemblies.

## Materials and methods

### DNA constructs for protein expression

The cDNAs for expression of human DYNC1LI1 (UniProt ID: Q9Y6G9; residues 388–523, 388–471, 402–471, 414–471, 424–471, 440–471, 440–455, 440–523, and 472–523) and human DYNC1LI2 (UniProt ID: O43237; residues 375–492, 375–450, and 451–492) were cloned into vector pGEX-6P-1 with a single N-terminal tryptophan and a C-terminal linker (GSGSG) followed by 6xHis. The cDNAs for human BICD2 (UniProt ID: Q8TD16; residues 2–422), RAB11FIP3 (UniProt ID: O75154; residues 2–756), HOOK3 (UniProt ID: Q86VS8; residues 2–239 and 2–552), NIN (UniProt ID: Q8N4C6; residues 1–693), SPDL1 (UniProt ID: Q96EA4; residues 2–359), and TRAK1 (UniProt ID: Q9UPV9; residues 103–167 and 103–187, with and without a C-terminal fusion to the GCN4 dimeric coiled-coil sequence VKQLEDKVEELLSKNAHLENEVARLKKLV [GCN4^CC^]) were cloned into a 2CT-derived vector containing an N-terminal 6xHis::MBP fusion followed by a linker with a Tobacco Etch Virus nuclear-inclusion-a endopeptidase (TEV protease) cleavage site and containing a C-terminal linker (GSGSGR) followed by the Strep-tag II. The cDNA of RILP (UniProt ID: Q96NA2; residues 1–401) was cloned into the pACEBac1 vector with a C-terminal linker (GSGSGR) followed by the Strep-tag II.

### Protein expression and purification from bacteria

All bacterial expression vectors were transformed into the *Escherichia coli* strain BL21, except for the NIN and HOOK3 constructs, which were transformed into the *E*. *coli* strain Rosetta. Expression was induced with 0.1 mM IPTG at 18°C overnight at an OD_600_ of 0.9, and cells were harvested by centrifugation for 20 min at 4,000*g*.

For GST::LIC::6xHis constructs used in pull-down experiments, bacterial pellets were resuspended in lysis buffer A (50 mM HEPES, 250 mM NaCl, 0.1% [v/v] Tween 20, 10 mM EDTA, 10 mM EGTA, 5 mM DTT, 1 mM phenylmethanesulfonyl fluoride [PMSF], 2 mM benzamidine-HCl, 1 mg/mL lysozyme [pH 8.0]), disrupted by sonication, and cleared by centrifugation at 34,000*g* for 45 min. GST::LIC::6xHis was purified by tandem affinity chromatography using glutathione agarose resin (Thermo Fisher Scientific) followed by HisPur Ni-NTA resin (Thermo Fisher Scientific). Glutathione agarose resin was incubated in batch with the cleared lysate and then washed with wash buffer A (25 mM HEPES, 250 mM NaCl, 0.1% Tween 20, 1 mM DTT, 2 mM benzamidine-HCl [pH 8.0]), and proteins were eluted on a gravity column with elution buffer A (50 mM HEPES, 150 mM NaCl, 10 mM reduced L-glutathione, 1 mM DTT, 2 mM benzamidine-HCl [pH 8.0]). Fractions containing the recombinant proteins were pooled, incubated in batch with Ni-NTA resin, and washed with wash buffer B (25 mM HEPES, 250 mM NaCl, 25 mM imidazole, 0.1% Tween 20, 1 mM DTT, 2 mM benzamidine-HCl [pH 8.0]). Proteins were eluted on a gravity column with elution buffer B (50 mM HEPES, 150 mM NaCl, 250 mM imidazole, 1 mM DTT, 2 mM benzamidine-HCl [pH 8.0]). Fractions containing the proteins were pooled and dialyzed against storage buffer (25 mM HEPES, 150 mM NaCl [pH 7.5]) or further purified by size-exclusion chromatography using a Superose 6 10/300 column (GE Healthcare) equilibrated with storage buffer. Glycerol and DTT were added to final concentrations of 10% (v/v) and 1 mM, respectively, and aliquots were flash-frozen in liquid nitrogen and stored at −80°C.

Purification of LIC1::6xHis (residues 388–523, 388–471, and 472–523) for NMR spectroscopy, SPR, and MST experiments was carried out as described above with the following modifications: GST::LIC1::6xHis was captured using a GSTrap FF column (GE Healthcare) and eluted with elution buffer A. The GST moiety was cleaved off in solution with PreScission Protease, glutathione was removed by dialysis (50 mM HEPES, 150 mM NaCl [pH 8.0]), and the sample was applied again to a GSTrap FF column to remove GST and GST-tagged Prescission Protease. The flow-through containing LIC1::6His was subjected to size-exclusion chromatography using a Superdex 75 increase 10/300 GL column (GE Healthcare) in NMR buffer (50 mM sodium phosphate, 150 mM NaCl [pH 6.5]).

For purification of cargo adaptors, bacterial pellets were resuspended in lysis buffer B (50 mM HEPES, 250 mM NaCl, 10 mM imidazole, 0.1% Tween 20, 1 mM DTT, 1 mM PMSF, 2 mM benzamidine-HCl, 1 mg/mL lysozyme [pH 8.0]), disrupted by sonication, and cleared by centrifugation at 34,000*g* for 45 min. The 6xHis::MBP::adaptor::Strep-tag II proteins were purified by tandem affinity chromatography using HisPur Ni-NTA resin followed by Strep-Tactin Sepharose resin (IBA). HisPur Ni-NTA resin was incubated in batch with the cleared lysate and then washed with wash buffer B, and proteins were eluted on a gravity column with elution buffer B. Fractions containing the recombinant proteins were pooled, incubated overnight with TEV protease to cleave off the 6xHis::MBP moiety (except for TRAK1 fragments, which were not cleaved), incubated in batch with Strep-Tactin Sepharose resin, and washed with wash buffer A. Proteins were eluted on a gravity column with elution buffer E (100 mM Tris-HCl, 150 mM NaCl, 1 mM EDTA, 2.5 mM desthiobiotin [IBA] [pH 8.0]). Fractions containing the proteins were pooled and dialyzed against storage buffer or further purified by size-exclusion chromatography using a Superose 6 10/300 column equilibrated with storage buffer. Glycerol and DTT were added to final concentrations of 10% and 1 mM, respectively, and aliquots were flash-frozen in liquid nitrogen and stored at −80°C.

### Protein expression and purification from insect cells

Bacmid recombination and virus production were performed as described previously [54]. A 500-mL culture (SFM4 medium; Hyclone) of Sf21 cells (0.8 × 10^6^ cells/mL) was infected with RILP::Strep-tag II-encoding virus. Cells were harvested by centrifugation at 800*g* for 5 min. Pellets were resuspended in lysis buffer C (50 mM HEPES, 250 mM NaCl, 1 mM DTT [pH 8.0]) supplemented with EDTA-free cOmplete Protease Inhibitor Cocktail (Roche), sonicated, and cleared by centrifugation at 34,000*g* for 45 min. RILP::Strep-tag II was purified by batch affinity chromatography using Strep-Tactin Sepharose. The resin was washed with wash buffer C (25 mM HEPES, 250 mM NaCl, 0.1% [v/v] Tween 20, 1 mM DTT [pH 8.0]), and the protein was eluted on a gravity column with elution buffer E. Fractions containing RILP::Strep-tag II were pooled and dialyzed against storage buffer. Glycerol and DTT were added to final concentrations of 10% and 1 mM, respectively, and aliquots were flash-frozen in liquid nitrogen and stored at −80°C.

### GST pull-down assays

Purified GST::LIC::6xHis constructs (50 pmol) were incubated with 250 pmol SPDL1(2–359)::Strep-tag II, 50 pmol BICD2(2–422)::Strep-tag II, 250 pmol NIN(1–693)::Strep-tag II, 50 pmol HOOK3(2–552)::Strep-tag II, 50 pmol RAB11FIP3(2–756)::Strep-tag II, 50 pmol RILP(1–401)::Strep-tag II, 250 pmol 6xHis::MBP::TRAK1(103–187)::Strep-tag II, 250 pmol 6xHis::MBP::TRAK1(103–167)::GCN4^CC^::Strep-tag II, or 250 pmol 6xHis::MBP::TRAK1(103–187)::GCN4^CC^::Strep-tag II for 1 h at 4°C in 150 μL pull-down buffer (50 mM HEPES, 100 mM NaCl, 5 mM DTT [pH 7.5]) containing 0.1% Tween 20 and supplemented with 15 μL of glutathione agarose resin. After washing the resin with 3 × 500 μL of the same buffer, proteins were eluted with pull-down buffer containing 15 mM reduced L-glutathione.

### Immunoblotting

For immunoblots of purified proteins, samples were resolved by 10% SDS-PAGE and transferred to 0.2-μm nitrocellulose membranes (GE Healthcare). Membranes were blocked in PBS (4 mM KH_2_PO_4_, 16 mM Na_2_HPO_4_, 115 mM NaCl [pH 7.4]) containing 3% (w/v) BSA and 0.5% (v/v) Tween 20 and probed at 4°C overnight with mouse StrepMAB-Classic antibody (IBA) at 1 μg/mL in PBS containing 0.2% BSA and 0.1% Tween 20. Membranes were washed three times with PBS/0.1% Tween 20 (PBST), incubated with goat anti-mouse antibody coupled to HRP (Jackson ImmunoResearch, 1:10,000) for 1 h at room temperature, and washed again three times with PBST. Proteins were visualized by chemiluminescence using Pierce ECL Western Blotting Substrate (Thermo Fisher Scientific) and X-ray film (Amersham, GE Healthcare).

For immunoblots of *C*. *elegans*, 100 adult hermaphrodites were collected into M9 buffer and processed for immunoblotting as described [37]. Samples were resolved on a gradient gel (4%–20%) and transferred to 0.2-μm nitrocellulose membranes. Membranes were blocked with 5% (w/v) nonfat dry milk in TBST (20 mM Tris, 140 mM NaCl, 0.1% Tween [pH 7.6]) and probed at 4°C overnight with rabbit anti-DHC-1 antibody GC4 (1:1,400, made in-house), mouse anti-FLAG M2 antibody (1:1,000, Sigma-Aldrich), or mouse anti-α-tubulin B512 antibody (1:5,000, Sigma-Aldrich). Membranes were sequentially rinsed 3× with TBST, 1× with 5% nonfat dry milk in TBST, and 3× with TBST. Membranes were incubated with goat secondary antibody coupled to HRP (Jackson ImmunoResearch, 1:10,000) for 1 h at room temperature and washed again 3× with TBST, 1× with 5% nonfat dry milk in TBST, and 3× with TBST. Proteins were visualized as described above.

### NMR spectroscopy

For backbone resonance assignment of LIC1(388–523)::6xHis, ^15^N-^1^H HSQC, HNCACB, CBCA(co)NH, HNCO, HN(ca)CO, and HNN spectra [55] were recorded on a triple-resonance Varian 900 NMR cryoprobe spectrometer at 25°C using a ^13^C/^15^N-labeled sample in 50 mM sodium phosphate and 150 mM NaCl at pH 6.5 and 375 μM protein concentration in a standard 5-mm Shigemi tube. The 3D spectra were acquired with a nonuniform sampling (NUS) scheme generated by the NUS@HMS scheme generator software [56] with 1,024 complex data points in the direct dimension and 30% sampling of the original 96 and 80 points in the indirect ^13^C and ^15^N dimension, respectively. The spectral widths were 14,045 Hz (^1^H), 3,200 Hz (^15^N), 3,770 Hz (C = O), and 15,835 Hz (Cα/Cβ); the interscan delay was 1.7 s; and the number of scans was 16 for all experiments. The NUS-acquired data were reconstructed using the hmsIST software [56]. Zero-filling was achieved by addition of 256 points in both indirect dimensions. A solvent subtraction function was applied in the direct dimension. Further data processing and visualization were performed using NMRPipe/NMRDraw [57] and NMRFAM Sparky [58]. Resonance assignment was performed using CCPNmr Analysis software [59].

Because of high sequence redundancy and extensive peak overlap, we used a “divide-and-conquer” approach for chemical shift assignment. We measured and overlaid ^15^N-^1^H HSQC spectra for two smaller LIC1 constructs, 388–471 and 472–523, with 388–523 to facilitate the verification of assignment. The ^15^N-^1^H HSQC spectra of the small constructs were measured with 128 scans and 128 complex points in the indirect dimension.

We assessed residual secondary structure using the SSP score program developed by Forman-Kay and colleagues with the re-referencing algorithm for ^13^CA and ^13^CB shifts [24]. The method combines different chemical shifts into a single residue–specific SSP score. Our input shifts were those of ^1^H^N^, ^15^N, ^13^CO, ^13^CA, and ^13^CB.

The ^15^N *R*_1_ and *R*_1ρ_ relaxation rate constants and ^15^N-^1^H heteronuclear NOEs of LIC1(388–523)::6xHis were measured on a Bruker 700 MHz spectrometer equipped with a triple-resonance cryoprobe at 25°C using a ^15^N-labeled sample at 400 μM protein concentration in 50 mM sodium phosphate buffer, 50 mM NaCl, 0.05% (w/v) NaN_3_, and 5 mM DTT at pH 6.5. For the *R*_1_ and *R*_1ρ_ experiments, the sampling time points were 40, 88, 136, 192, 288, 392, 592, 688, 792, and 992 ms and 30, 60, 120, 150, 180, and 210 ms, respectively. During the *R*_1ρ_ relaxation time, a ^15^N spin-lock field of 1,433-Hz strength was applied. *R*_2_ was calculated from *R*_1_ and *R*_1*ρ*_ using the following equation: *R*_2_ = *R*_1ρ_ + (*R*_1ρ_–*R*_1_)*tg^2^(θ), where θ = tan^-1^(2πΔν/γ_N_B_1_), Δν is the resonance offset, |γ_N_B_1_|/2π is the strength of the spin-lock field B_1_, and γ_N_ the gyromagnetic ratio of the ^15^N spin. The ^15^N-^1^H heteronuclear NOEs were determined from two spectra recorded in presence and in the absence of ^1^H saturation in an interleaved manner.

For NMR titration analysis, ^15^N-^1^H HSQC spectra with 128 complex points in the indirect dimension and 128 scans were recorded on a triple-resonance Varian 900 NMR cold-probe spectrometer at 25°C of 40 μM samples of ^15^N-labeled LIC1(388–523)::6xHis alone and in the presence of 0.5 and 1 equivalent of unlabeled SPDL1(2–359)::Strep-tag II, BICD2(2–422)::Strep-tag II, or HOOK3(2–239)::Strep-tag II in 50 mM sodium phosphate and 150 mM NaCl at pH 6.5. We used freshly prepared samples for each titration step to limit confusion with potential degradation peaks. Data processing and visualization were performed using NMRPipe/NMRDraw [57] and NMRFAM Sparky [58].

The ^1^H, ^13^C, and ^15^N chemical shift assignments have been deposited in the BioMagResBank database (http://www.bmrb.wisc.edu) under the accession number 27401.

### SPR

SPR analysis with SPDL1, BICD2, and HOOK3 fragments was conducted with a Biacore 3000 system. His-tagged LIC1(388–523) and LIC1(388–471) constructs (ligand) were immobilized on three different flow cells (FC1–3) on an NTA sensor chip at densities of 200–300 RU, whereas the fourth flow cell (FC4) was spared for blank sensogram measurement. Concentrated stocks of Strep-tagged SPDL1(2–359), BICD2(2–422), and HOOK3(2–239) (analyte) were dialyzed exhaustively against HBS-P flow buffer (10 mM HEPES, 150 mM NaCl, 0.005% [v/v] surfactant P_20_ [pH 7.4]). The background-blank sensogram was subtracted from sensograms measured with immobilized ligands. Injections for each analyte concentration were performed in triplicate. Data processing was done on Biacore evaluation software.

SPR analysis with RILP::Strep-tag II was performed using a Biacore X100 system equipped with a CM5 sensor chip (GE Healthcare). Anti-GST antibody was immobilized using amine-coupling chemistry using the Amine Coupling Kit (GE Healthcare) and the GST Capture Kit (GE Healthcare) according to manufacturer's instructions. The surfaces of flow cells 1 and 2 were activated for 7 min with a 1:1 mixture of 0.1 M N-hydroxysuccinimide and 0.4 M 1-ethyl-3-(3-dimethylaminopropyl)carbodiimide at a flow rate of 5 μL/min. Anti-GST antibody at a concentration of 30 μg/mL in 10 mM sodium acetate (pH 5.0) was immobilized at a density of 7,500 RU on flow cells 1 and 2. Surfaces were blocked with a 7-min injection of 1 M ethanolamine (pH 8.0). Anti-GST antibody high-affinity sites were blocked with 3 cycles of a 3-min injection of recombinant GST at 5 μg/mL in running buffer (10 mM HEPES, 150 mM NaCl, 3 mM EDTA, 0.05% surfactant P_20_ [pH 7.4]) followed by a 2-min injection of regeneration solution (10 mM glycine-HCl [pH 2.1]). To collect kinetic binding data, at the beginning of each cycle, GST::LIC1(388–523)::6xHis or GST::LIC1(440–455)::6His in running buffer (ligand) was injected over flow cell 2 at a density of 720–530 and 550–460 RU, respectively. Flow cell 1 was injected with GST::6xHis at a density of 575–430 RU to serve as a reference surface. RILP::Strep-tag II (analyte) was injected in running buffer over the two flow cells at concentrations of 50, 11, 4.5, 1.8, and 0.3 μM at a flow rate of 30 μL/min and a temperature of 25°C. The complex was allowed to associate and dissociate for 120 and 600 s, respectively. Surfaces were regenerated with two 2-min injections of regeneration solution. Three independent runs were performed for each condition. The data were fit to a 1:1 interaction model using the evaluation module of the Biacore X100 software, version 2.0.1 (GE Healthcare).

### MST

Measurements were carried out on a NanoTemper Monolith NT.115 pico instrument (NanoTemper Technologies) at 25°C using medium power and 20% excitation power (auto-detect-pico-red). LIC1(388–523)::6xHis was fluorescently labeled by interacting 100 μL protein solution (200 nM concentration) with 100 μL Red-Tris NTA dye (100 nM concentration). The reaction mixture was incubated at room temperature for 30 min followed by centrifugation for 10 min at 4°C with 15,000*g*. Then, 10 nM of labeled LIC1(388–523)::6xHis and 16 two-fold dilution series of the adaptor were loaded into 16 standard capillaries (NanoTemper Technologies) (SPDL1[2–359]::Strep-tag II highest concentration 42.5 μM; BICD2[2–422]::Strep-tag II highest concentration 36.5 μM; HOOK3[2–239]::Strep-tag II highest concentration 70 μM). We observed sigmoidal behavior of the fluorescence level over time, which allowed characterization of the interactions. Raw data were analyzed using the NanoTemper software (MO affinity analysis v 2.2.7). The signal-to-noise ratios for SPDL1(2–359)::Strep-tag II, BICD2(2–422)::Strep-tag II, and HOOK3(2–239)::Strep-tag II were 18.8, 22.1, and 7.1, respectively.

### CD

CD spectra of LIC1(388–523)::6xHis were collected on a J-815 CD spectrometer (Jasco) with a wavelength range from 190 to 250 nm, data pitch 1 nm, standard sensitivity, 1 nm bandwidth and 20 nm/min scanning speed, at temperatures of 25, 37, 50, 60, and 70°C. The baseline of the spectrum was obtained from measurement of the buffer (50 mM sodium phosphate, 150 mM NaCl [pH 6.5]) and subtracted from the spectra of the samples to remove artificial CD signals that might originate from the optical system. Data measurement and analysis were performed using Spectra Manager version 2 (Jasco). Molar ellipticity was calculated as *m°M*/(10*LC*), where *m°* is the degree value (mdeg), *M* is the molecular weight in g/mole, *L* is the path length in cm, and *C* is the concentration in g/L.

### *C*. *elegans* strains

Worm strains (S1 Table) were maintained at 20°C on standard nematode growth media (NGM) plates seeded with OP50 bacteria. A Mos1 transposon-based strategy (MosSCI) was used to generate strains stably expressing mKate2::RAB-5, SNB-1::mKate2, and TOMM-20(1–54)::mKate2 in touch receptor neurons [33]. Transgenes were cloned into pCFJ151 for insertion on Chromosome 2 (ttTi5605 locus), and transgene integration was confirmed by PCR. C-terminal mutants of *dli-1* (Δ369–443, F392A/F393A, L396A/L397A, and Δ414–443) and *3xflag*::*dli-1* were generated by CRISPR/Cas9-mediated genome editing, as described previously [60,61]. Genomic sequences targeted by sgRNAs are listed in S2 Table. Modifications in genomic DNA sequence were confirmed by sequencing, and strains were outcrossed 6 times against the wild-type N2 strain to remove potential background mutations. Other fluorescent markers were subsequently introduced by mating. The *dli-1* mutants Δ369–443, F392A/F393A, and L396A/L397A were maintained as heterozygotes using the GFP-marked genetic balancer nT1 [qIs51]. Homozygous F1 progeny from balanced heterozygous mothers were identified by the absence of GFP fluorescence.

### Life span assay

Animals were collected at the L4 stage (day 0) and transferred every 2 d to a new NGM plate with bacteria. Animals were scored as alive or dead every 1–3 d. Animals were considered dead if they did not respond when touched with a platinum wire and if there was no evidence of pharyngeal pumping. Animals that were found dead on the edge of the plate, escaped, or died because of internal hatching of progeny were excluded from the assay.

### Imaging

#### Body bending assay

L4 hermaphrodites were transferred to a new NGM plate with bacteria 16 h before performing the assay. For imaging, animals were transferred to a slide containing a 2-μL drop of M9. Movements were tracked at 20°C for 1 min at 40 frames per second using an SMZ 745T stereoscope (Nikon) mounted with a QIClic CCD camera (QImaging) and controlled by Micro-Manager software (Open Imaging). The wrMTrck plugin for Image J (http://www.phage.dk/plugins/wrmtrck.html) was used for automated counting of body-bends.

#### Differential interference contrast imaging of adults animals

L4 hermaphrodites were transferred to a new NGM plate with bacteria for 16 h, paralyzed with 50 mM of sodium azide for 5 min, mounted on a freshly prepared 2% (w/v) agarose pad, and covered with an 18 mm × 18 mm coverslip (No. 1.5H, Marienfeld). Imaging was performed on an Axio Observer microscope (Zeiss) equipped with an Orca Flash 4.0 camera (Hamamatsu) controlled by ZEN 2.3 software (Zeiss). Multiple images covering the entire animal were recorded at 1 × 1 binning with a 40x NA 1.3 Plan-Neofluar objective and assembled into one image using the tiles mode in ZEN.

#### Touch receptor neurons

To assess the axonal distribution of mKate2::RAB-5, SNB-1::mKate2, and TOMM-20(1–54)::mKate2, hermaphrodites at the day 1 adult stage were paralyzed with 50 mM of sodium azide for 5 min, transferred to a 2% agarose pad, and imaged on the Axio Observer microscope described above using an HXP 200C Illuminator (Zeiss). For mKate2::RAB-5 and SNB-1::mKate2, z-stacks (1-μm z steps) of the axonal tip and the nerve ring in ALM and AVM neurons (marked by soluble GFP) were acquired at 1 × 1 binning with a 63x NA 1.4 Plan-Apochromat objective. For TOMM-20(1–54)::mKate2, z-stacks capturing the entire ALM neuron were recorded at 1 × 1 binning with a 40x NA 1.3 Plan-Neofluar objective.

For live imaging of mKate2::RAB-5 and TOMM-20(1–54)::mKate2, hermaphrodites at the L4 stage were paralyzed with 5 mM levamisole in M9 buffer for 10 min and mounted onto freshly prepared 2% or 5% agarose pads, respectively. ALM neurons were identified using the GFP signal, and only neurons with morphologically healthy axons were imaged. Time-lapse sequences of an axonal region approximately 50 μm away from the cell soma were recorded in a temperature-controlled room at 20°C, using a Nikon Eclipse Ti microscope coupled to an Andor Revolution XD spinning disk confocal system, composed of an iXon Ultra 897 CCD camera (Andor Technology), a solid-state laser combiner (ALC-UVP 350i, Andor Technology), and a CSU-X1 confocal scanner (Yokogawa Electric Corporation) controlled by Andor iQ3 software (Andor Technology). For mKate2::RAB-5, a single image was acquired every 200 ms for a total of 30 s at 1 × 1 binning using a 100x NA 1.45 Plan-Apochromat objective. For TOMM-20(1–54)::mKate2, a 5 × 0.5 μm z-stack was acquired every 5 s for a total of 5 min at 1 × 1 binning using a 60x NA 1.4 Plan-Apochromat objective.

#### One-cell embryos

Adult gravid hermaphrodites were dissected in a watch glass filled with a 0.7× dilution of Egg Salts medium (1× medium is 118 mM NaCl, 40 mM KCl, 3.4 mM MgCl_2_, 3.4 mM CaCl_2_, 5 mM HEPES [pH 7.4]), and embryos were mounted on a 2% agarose pad. Imaging was performed using the spinning disk confocal system described above at 1 × 1 binning using an 60x NA 1.4 Plan-Apochromat objective. A 12 × 1 μm z-stack of mCherry::HIS-11 (histone H2B) and GFP::TBB-2 (β-tubulin) was acquired every 10 s from the start of pronuclear migration until the onset of cytokinesis.

### Image analysis

Image analysis was performed using Fiji software (Image J version 1.52d).

#### mKate2::RAB-5 and SNB-1::mKate2 levels

For axonal tip measurements, the GFP signal was used as a reference to draw a region around the last 20 μm of the ALM or AVM axon, and the integrated intensity of mKate2 fluorescence within that region was measured. The region was then expanded by a few pixels along the length of the axonal segment. The difference in integrated fluorescence intensity between the outer and inner region was used to define the integrated background intensity after normalization to the area of the inner region. The final integrated intensity of mKate2 signal at the axonal tip was then calculated by subtracting the integrated background intensity from the integrated intensity of the inner region. The same approach was used to determine integrated mKate2 intensity in the nerve ring.

#### TOMM-20(1–54)::mKate2 distribution

To determine the number and position of mitochondria along the ALM axon (marked by GFP), line segments connecting adjacent TOMM-20(1–54)::mKate2 particles were drawn with the first segment starting at the cell body and the last segment terminating at the axonal tip. The sum of the lengths of individual line segments was equal to the total length of the axon. The distance of each TOMM-20(1–54)::mKate2 particle from the cell body was then normalized to axon length (i.e., 0% corresponds to the beginning of the axon at the cell body and 100% denotes the axonal tip).

#### Axonal transport parameters

Only time-lapse sequences during which there was no discernable movement of the animal and that had uniformly bright mKate2 signal along the axonal segment were considered. Kymograph generation and image analysis were performed with KymoAnalyzer, a semiautomated open-source ImageJ package of macros designed to quantify transport parameters of fluorescently labeled stationary or moving particles [62]. A track is defined as a single particle trajectory, and a segment corresponds to a portion within a track of an individual moving particle framed by a pause or a reversal. Values for run length and velocity reported in this study are for segments. To determine the fraction of particles moving in the anterograde or retrograde direction, the position of the start and end point of the track was considered (i.e., a net anterograde moving particle may also have one or more segments of retrograde movement along its track and vice-versa; a net stationary particle may have one or more segments of anterograde or retrograde movement along its track).

#### Centrosome–centrosome axis tilt in the one-cell embryo

After maximum intensity projection of z-stacks, the angle between the centrosome–centrosome axis and anterior–posterior axis at NEBD was calculated from the coordinates of the two centrosomes and the two outermost points along the embryo long axis, using the cytoplasmic GFP signal to visualize the embryo outline.

### Statistical analysis

Statistical analysis was performed with GraphPad Prism 7.0 software. Values in figures and text are reported as mean ± SEM. The type of statistical analysis performed is described in each figure legend. Differences were considered significant at *P* < 0.05.

## Supporting information

S1 Fig Sequence alignment of the LIC C-terminal region and structural features of seven cargo adaptors with diverse functions. Multiple sequence alignment of the LIC C-terminal region in vertebrate and invertebrate species, including commonly used model organisms. The position of the conserved helical segments 1 and 2 is indicated above the human LIC1 sequence. (B) Domain architecture of the human cargo adaptors examined in this study: BICD2 (UniProt ID: Q8TD16), SPDL1 (UniProt ID: Q96EA4), HOOK3 (UniProt ID: Q86VS8), RILP (UniProt ID: Q96NA2), RAB11FIP3 (UniProt ID: O75154), NIN (UniProt ID: Q8N4C6), and TRAK1 (UniProt ID: Q9UPV9). For NIN, only the first 1,000 of 2,090 residues are represented. Predictions of α-helical and coiled-coil segment were performed using the JPred Secondary Structure Prediction server. Protein domains (Hook, RILP homology 1 and 2, proline rich, EF-hand) are shown as annotated in the UniProt database. The CC1 box, which is required for the interaction with LIC in BICD2, SPDL1, and presumably TRAK1, is also highlighted. Dashed rectangles indicate the recombinant purified protein fragments used for in vitro assays. For HOOK3, fragment 2–552 was used for GST pull-downs experiments in Fig 3 and Fig 4. For NMR (Fig 2D), SPR (Fig 2G), and MST (S5C Fig) experiments, which required more protein, we used HOOK3(2–239), because we obtained a higher yield from bacteria with this fragment than with HOOK3(2–552). HOOK3(2–239) had previously been shown to be sufficient for LIC binding [17]. BICD2, bicaudal D homolog 2; GST, glutathione S-transferase; HOOK3, Hook homolog 3; LIC, light intermediate chain; MST, microscale thermophoresis; NIN, ninein; NMR, nuclear magnetic resonance; RAB11FIP3, RAB11 family-interacting protein 3; RILP, RAB-interacting lysosomal protein; SPDL1, Spindly; SPR, surface plasmon resonance; TRAK1, trafficking kinesin-binding protein 1.
(TIF)

S2 Fig Backbone resonance assignment of LIC1(388–523) and lack of long-range interactions between N- and C-terminal segments. (A) Overlay of ^15^N-^1^H HSQC spectra of LIC1(388–523)::6xHis (red) and the two smaller constructs LIC1(388–471)::6xHis (green) and LIC1(472–523)::6xHis (magenta). (B) Two F1-F2 strips showing ^15^N-^15^N correlations through the 3D HNN spectrum at the F3-^1^H chemical shift of two LIC1-C residues. Off-diagonal peaks (orange) indicate the ^15^N chemical shift of the residues preceding and succeeding the residue represented by the diagonal peak (blue). Shown are the connectivities of S516 and M434. HNN, ^1^H-^15^N-^15^N correlation; HSQC, heteronuclear single quantum coherence; LIC, light intermediate chain; LIC1-C, C-terminal light intermediate chain 1.
(TIF)

S3 Fig *R*_1ρ_ and *R*_1_ relaxation rate constants and ^15^N-^1^H heteronuclear nuclear Overhauser enhancements with experimental errors. (A–C) *R*_1ρ_, *R*_1_ relaxation rates constants and heteronuclear Overhauser enhancements of LIC1(388–523) at 700 MHz are shown in (A), (B), and (C), respectively. The error bars indicate the standard deviations obtained from Monte Carlo simulations with 100 runs and normally distributed uncertainties based on the spectral noise added to each peak intensity. *R*_2_ shown in Fig 1E is calculated from *R*_1ρ_ and *R*_1_. Underlying data for S3 Fig can be found in S1 Data. LIC1, light intermediate chain 1.
(TIF)

S4 Fig CD spectra of SPDL1(2–359) and LIC1(388–523). (A) The spectrum of SPDL1(2–359)::Strep-tag II at 25°C indicates α-helical secondary structure with a molar ellipticity of 1.05. (B) Spectra of LIC1(388–523)::6xHis suggest the presence of α-helical propensity over a wide temperature range (25–70°C). Underlying data for S4 Fig can be found in S1 Data. CD, circular dichroism; LIC1, light intermediate chain 1; SPDL1, Spindly.
(TIF)

S5 Fig MST analysis of the interaction between LIC1(388–523) and N-terminal regions of SPDL1, BICD2, and HOOK3. (A–C) MST assays with Strep II–tagged SPDL1(2–359) (A), BICD2(2–422) (B), and HOOK3(2–239) (C) yield the indicated fitted *K*_D_ values (mean ± SEM). Sixteen serial dilutions of every adaptor were mixed with fluorescently labeled LIC1(388–523) in standard capillary tubes. Underlying data for S5 Fig can be found in S1 Data. BICD2, bicaudal D homolog 2; HOOK3, Hook homolog 3; LIC1, light intermediate chain 1; MST, microscale thermophoresis; SPDL1, Spindly.
(TIF)

S6 Fig C-terminal LIC1 helix 1 binds to RILP with similar affinity as the entire LIC1 C-terminal region. (A, B) Sensogram (black lines) and corresponding 1:1 fitting (red lines) of the interaction between GST::LIC1(388–523)::6xHis (A) or GST::LIC1(440–455)::6xHis (B) with RILP::Strep-tag II. One of three replicates is shown. GST::LIC1 constructs were immobilized on the sensor chip with an anti-GST antibody. Fitted constants (mean ± SEM): *K*_*a*_ = 8.72 ± 0.83 × 10^2^ M^−1^ s^−1^ and *K*_*d*_ = 10.10 ± 0.46 × 10^−4^ s^−1^, which results in *K*_*D*_ = 1.18 ± 0.14 × 10^−6^ M for LIC1(388–523) (A); *K*_*a*_ = 6.06 ± 0.31 × 10^2^ M^−1^ s^−1^ and *K*_*d*_ = 9.96 ± 0.72 × 10^−4^ s^−1^, which results in *K*_*D*_ = 1.67 ± 0.21 × 10^−6^ M for LIC1(440–455) (B). Underlying data for S6 Fig can be found in S1 Data. BICD2, bicaudal D homolog 2; GST, glutathione S-transferase; LIC 1, light intermediate chain 1; RILP, RAB-interacting lysosomal protein.
(TIF)

S7 Fig *C*. *elegans* DLI-1 is required for the stability of DHC-1. (A) Immunoblot of adult hermaphrodites expressing endogenously tagged 3xFLAG::DLI-1, showing that depletion of DLI-1 by RNAi reduces DHC-1 levels. α-Tubulin serves as the loading control. (B) Immunoblot comparing DHC-1 levels in wild-type and *dli-1(Δ369–443)* animals with those in animals expressing endogenous DHC-1 tagged with GFP. α-Tubulin serves as the loading control. Note that GFP-tagged DHC-1 is expressed at higher levels than DHC-1 in either wild-type or *dli-1(Δ369–443)* animals. Since DHC-1::GFP animals do not exhibit any obvious defects [37], the slight increase in DHC-1 levels in *dli-1* mutants relative to wild-type animals is unlikely to be the cause for the phenotype. DHC-1, dynein heavy chain 1; GFP, green fluorescent protein; RNAi, RNA interference.
(TIF)

S8 Fig Effect of C-terminal *dli-1* mutants on axonal transport. (A) Quantification of early endosome motility (mKate2::RAB-5), based on the analysis of kymographs as shown in Fig 5F. Graphs represent the mean ± SEM. For time spent in anterograde motion, retrograde motion, or pause, *n* represents the total number of tracks. For pause duration, *n* reflects the total number of segments within tracks of moving particles framed by a pause or a reversal. Results are derived from 2–5 independent imaging sessions. Statistical significance (mutant versus WT *dli-1*) was determined by one-way ANOVA on ranks (Kruskal-Wallis nonparametric test) followed by Dunn's multiple comparison test. ****P < 0.0001; *P < 0.05; ns indicates *P* > 0.05. (B) Cartoon showing the axonal tips imaged in animals coexpressing the synaptic vesicle marker SNB-1::mKate2 and soluble GFP in touch receptor neurons. (C) Fluorescence images of axonal tips in day 1 adults, showing misaccumulation of synaptic vesicles in the *dli-1(L396A/L397A)* mutant. Scale bar, 10 μm. (D) Quantification of synaptic vesicle misaccumulation in axonal tips using fluorescence intensity measurements of SNB-1::mKate2 as shown in (C). Graph represents the mean ± SEM signal in A.U. for *n* number of neurons imaged in two independent experiments. Statistical significance was determined with the Mann-Whitney test. ****P < 0.0001. (E, F) Quantification of mitochondrial motility (TOMM-20[1–54]::mKate2), based on the analysis of kymographs as shown in Fig 7F. Graphs represent the mean ± SEM. The total number *n* of axons (E) or segments (F) is indicated. Results are derived from 2–5 independent imaging sessions. Statistical significance was determined with the Mann-Whitney test. ns indicates *P* > 0.05. Underlying data for S8 Fig can be found in S1 Data. A.U., arbitrary units; GFP, green fluorescent protein; ns, not significant; SNB-1, synaptobrevin 1; WT, wild type.
(TIF)

S1 Movie Axonal transport of mKate2::RAB-5 in *dli-1(wild type)*, *dli-1(Δ369–443)*, *dli-1(F392A/F393A)*, and *dli-1(L396A/L397A)* animals at the L4 stage. Cell body of the ALM neuron is to the right. A single confocal section was acquired every 0.2 s. Playback speed is 30 frames per second. Scale bar, 10 μm. ALM, anterior lateral mechanosensory.
(MOV)

S2 Movie Axonal transport of TOMM-20(1–54)::mKate2 in *dli-1(wild type)* and *dli-1(L396A/L397A)* animals at the L4 stage. Cell body of the ALM neuron is to the right. A 5 × 0.5-μM z-stack was acquired every 5 s. Movie is a maximum intensity projection of z-stacks. Playback speed is 10 frames per second. Scale bar, 10 μm. ALM, anterior lateral mechanosensory; TOMM-20, translocase of outer mitochondrial membrane 20.
(MOV)

S1 Table *C*. *elegans* strains. (DOCX)

S2 Table Sequences targeted by sgRNAs for CRISPR/Cas9-mediated genome editing. CRISPR/Cas9, clustered regularly interspaced short palindromic repeat/CRISPR-associated 9; sgRNA, single guide RNA.
(DOCX)

S1 Data Values used to generate graphs. (XLSX)

## Abbreviations

ALM: anterior lateral mechanosensory
AVM: anterior ventral mechanosensory
ARP1: actin-related protein 1
BICD2: bicaudal D homolog 2
BICDR1: BICD-related protein 1
CC1: first coiled-coil segment
CD: circular dichroism
CRISPR/Cas9: clustered regularly interspaced short palindromic repeat/CRISPR-associated 9
DHC-1: dynein heavy chain 1
DLI-1: dynein light intermediate chain 1
dynein: cytoplasmic dynein 1
EM: electron microscopy
GCN4: general control nondepressible 4
GFP: green fluorescent protein
GST: glutathione S-transferase
HOOK3: Hook homolog 3
HSQC: heteronuclear single quantum coherence
HC: heavy chain
IC: intermediate chain
LC: light chain
LIC: light intermediate chain
LIC-C: C-terminal region of the LIC subunit
MBP: maltose-binding protein
MIRO: mitochondrial Rho
MST: microscale thermophoresis
NEBD: nuclear envelope breakdown
NIN: ninein
NMR: nuclear magnetic resonance
ns: not significant
ppm: parts per million
NTA: nitrilotriacetic acid
RAB11FIP3: RAB11 family-interacting protein 3
RILP: RAB-interacting lysosomal protein
RNAi: RNA interference
SNB-1: synaptobrevin 1
SPDL1: Spindly
SPR: surface plasmon resonance
SSP: secondary structure propensity
TOMM-20: translocase of outer mitochondrial membrane 20
TRAK1: trafficking kinesin-binding protein 1
