# Supplementary figures and images for: A transient helix in the disordered region of dynein light intermediate chain links the motor to structurally diverse adaptors for cargo transport
Source: PLoS Biol. 2019 Jan 7;17(1):e3000100. doi: 10.1371/journal.pbio.3000100 (PMC6336354; doi:10.1371/journal.pbio.3000100)

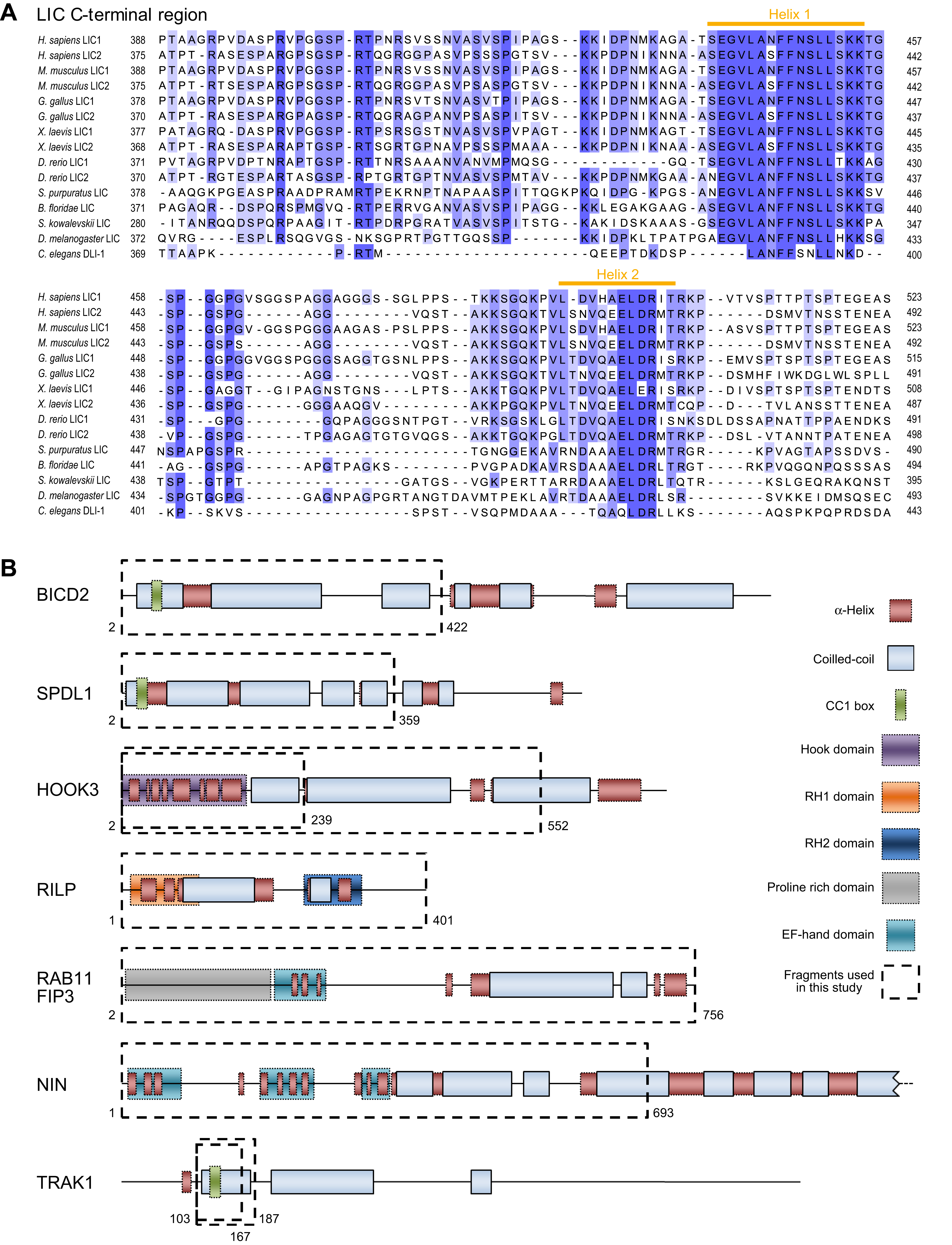

Supplement: S1 Fig — Multiple sequence alignment of the LIC C-terminal region in vertebrate and invertebrate species, including commonly used model organisms. The position of the conserved helical segments 1 and 2 is indicated above the human LIC1 sequence. (B) Domain architecture of the human cargo adaptors examined in this study: BICD2 (UniProt ID: Q8TD16), SPDL1 (UniProt ID: Q96EA4), HOOK3 (UniProt ID: Q86VS8), RILP (UniProt ID: Q96NA2), RAB11FIP3 (UniProt ID: O75154), NIN (UniProt ID: Q8N4C6), and TRAK1 (UniProt ID: Q9UPV9). For NIN, only the first 1,000 of 2,090 residues are represented. Predictions of α-helical and coiled-coil segment were performed using the JPred Secondary Structure Prediction server. Protein domains (Hook, RILP homology 1 and 2, proline rich, EF-hand) are shown as annotated in the UniProt database. The CC1 box, which is required for the interaction with LIC in BICD2, SPDL1, and presumably TRAK1, is also highlighted. Dashed rectangles indicate the recombinant purified protein fragments used for in vitro assays. For HOOK3, fragment 2–552 was used for GST pull-downs experiments in Fig 3 and Fig 4. For NMR (Fig 2D), SPR (Fig 2G), and MST (S5C Fig) experiments, which required more protein, we used HOOK3(2–239), because we obtained a higher yield from bacteria with this fragment than with HOOK3(2–552). HOOK3(2–239) had previously been shown to be sufficient for LIC binding [17]. BICD2, bicaudal D homolog 2; GST, glutathione S-transferase; HOOK3, Hook homolog 3; LIC, light intermediate chain; MST, microscale thermophoresis; NIN, ninein; NMR, nuclear magnetic resonance; RAB11FIP3, RAB11 family-interacting protein 3; RILP, RAB-interacting lysosomal protein; SPDL1, Spindly; SPR, surface plasmon resonance; TRAK1, trafficking kinesin-binding protein 1. (TIF) [file pbio.3000100.s001.tif]

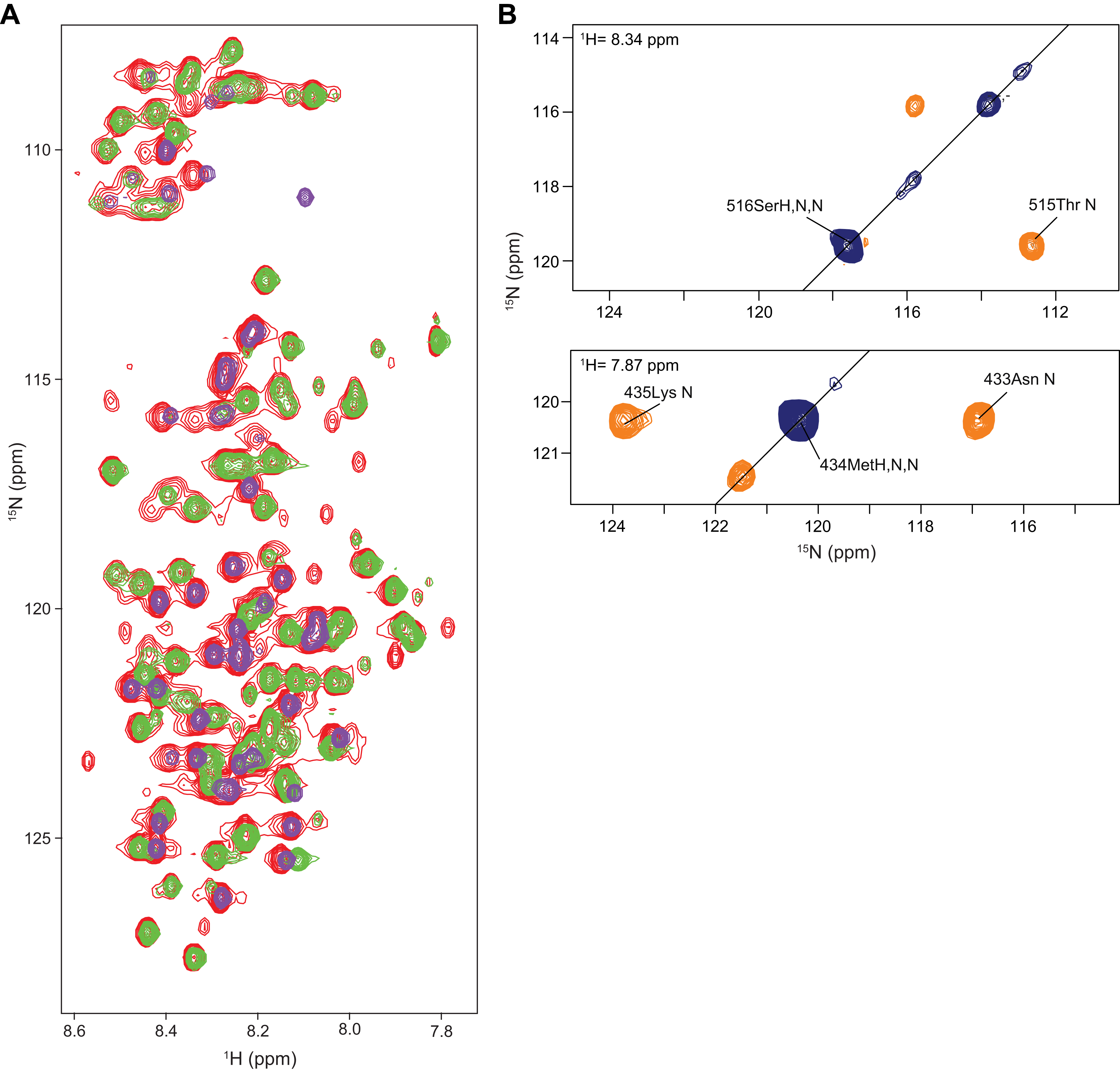

Supplement: S2 Fig — (A) Overlay of 15N-1H HSQC spectra of LIC1(388–523)::6xHis (red) and the two smaller constructs LIC1(388–471)::6xHis (green) and LIC1(472–523)::6xHis (magenta). (B) Two F1-F2 strips showing 15N-15N correlations through the 3D HNN spectrum at the F3-1H chemical shift of two LIC1-C residues. Off-diagonal peaks (orange) indicate the 15N chemical shift of the residues preceding and succeeding the residue represented by the diagonal peak (blue). Shown are the connectivities of S516 and M434. HNN, 1H-15N-15N correlation; HSQC, heteronuclear single quantum coherence; LIC, light intermediate chain; LIC1-C, C-terminal light intermediate chain 1. (TIF) [file pbio.3000100.s002.tif]

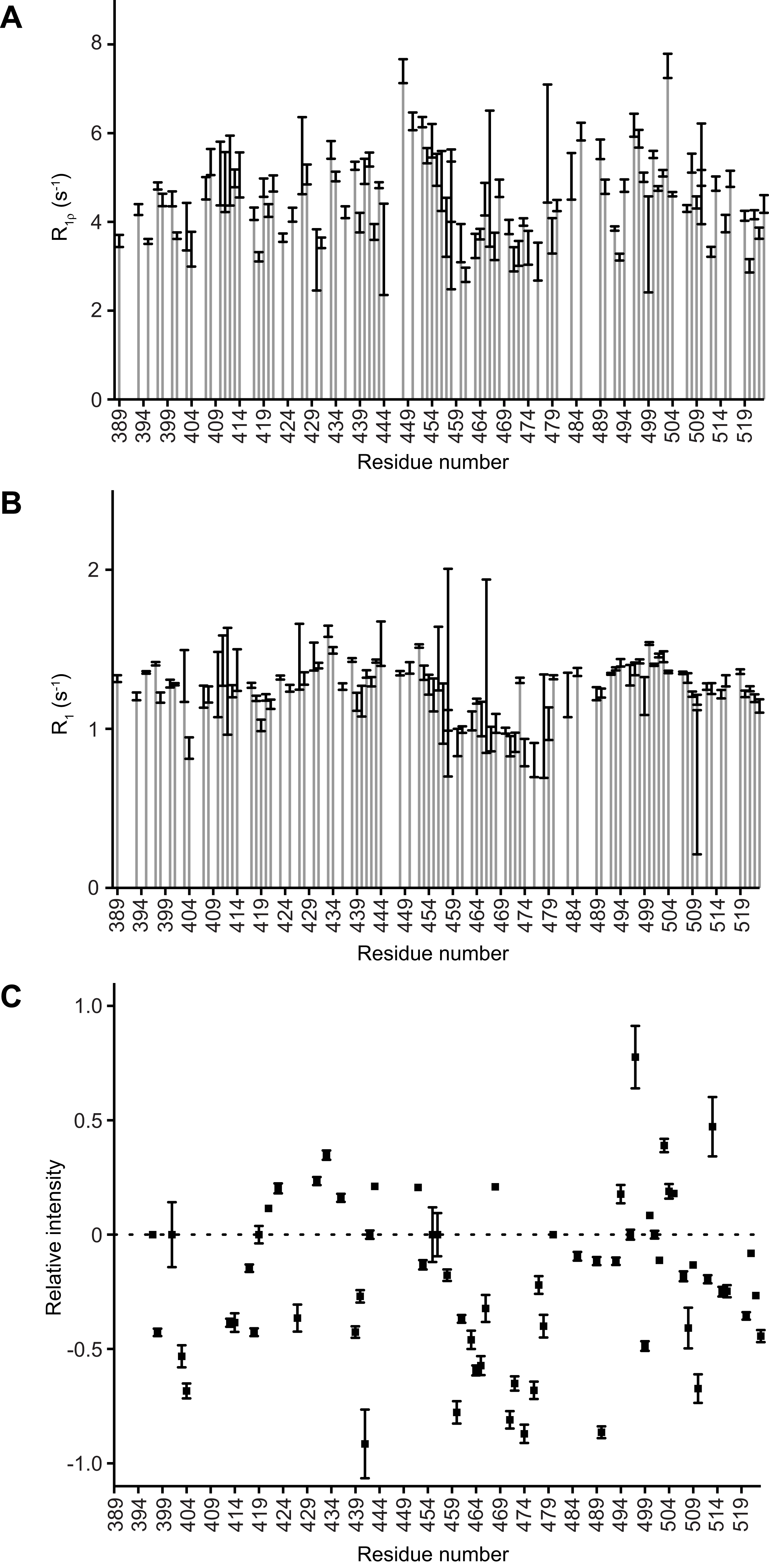

Supplement: S3 Fig — (A–C) R1ρ, R1 relaxation rates constants and heteronuclear Overhauser enhancements of LIC1(388–523) at 700 MHz are shown in (A), (B), and (C), respectively. The error bars indicate the standard deviations obtained from Monte Carlo simulations with 100 runs and normally distributed uncertainties based on the spectral noise added to each peak intensity. R2 shown in Fig 1E is calculated from R1ρ and R1. Underlying data for S3 Fig can be found in S1 Data. LIC1, light intermediate chain 1. (TIF) [file pbio.3000100.s003.tif]

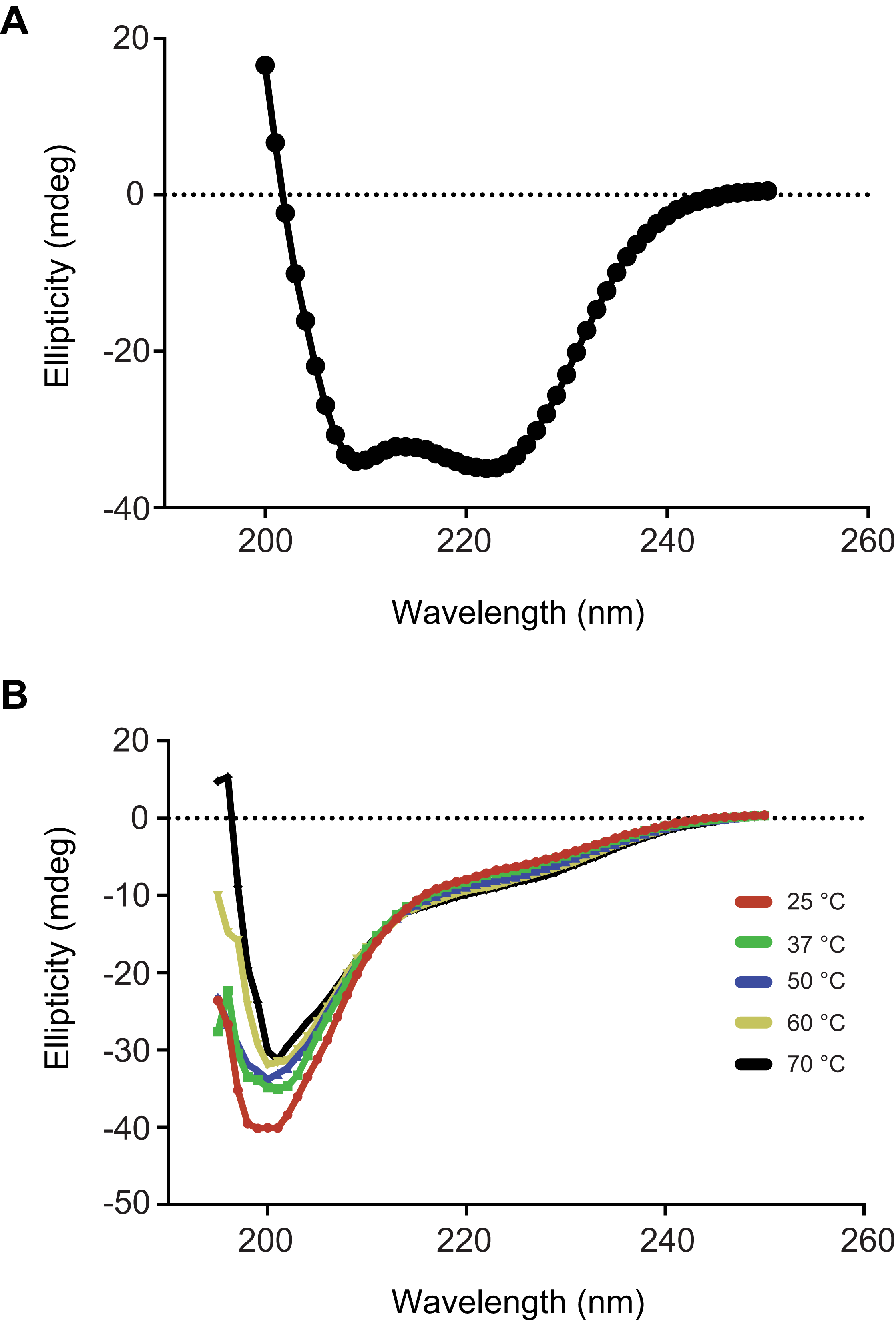

Supplement: S4 Fig — (A) The spectrum of SPDL1(2–359)::Strep-tag II at 25°C indicates α-helical secondary structure with a molar ellipticity of 1.05. (B) Spectra of LIC1(388–523)::6xHis suggest the presence of α-helical propensity over a wide temperature range (25–70°C). Underlying data for S4 Fig can be found in S1 Data. CD, circular dichroism; LIC1, light intermediate chain 1; SPDL1, Spindly. (TIF) [file pbio.3000100.s004.tif]

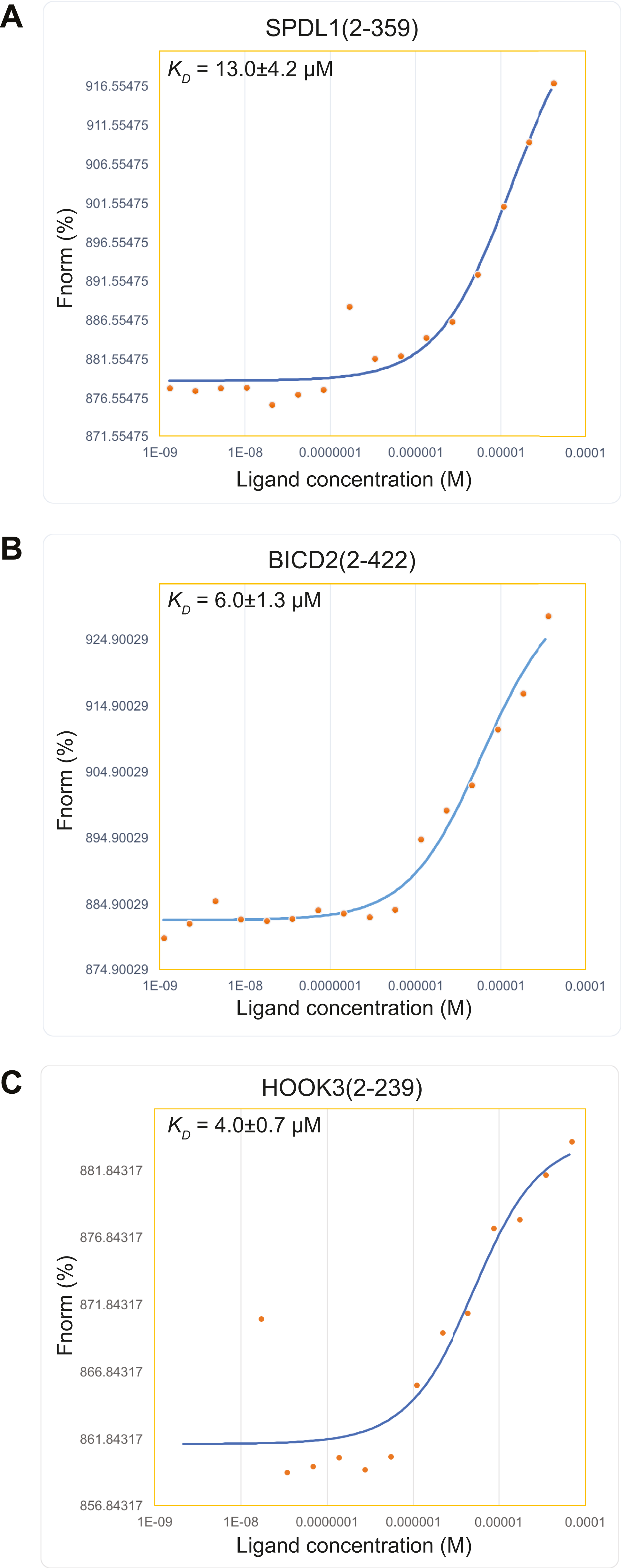

Supplement: S5 Fig — (A–C) MST assays with Strep II–tagged SPDL1(2–359) (A), BICD2(2–422) (B), and HOOK3(2–239) (C) yield the indicated fitted KD values (mean ± SEM). Sixteen serial dilutions of every adaptor were mixed with fluorescently labeled LIC1(388–523) in standard capillary tubes. Underlying data for S5 Fig can be found in S1 Data. BICD2, bicaudal D homolog 2; HOOK3, Hook homolog 3; LIC1, light intermediate chain 1; MST, microscale thermophoresis; SPDL1, Spindly. (TIF) [file pbio.3000100.s005.tif]

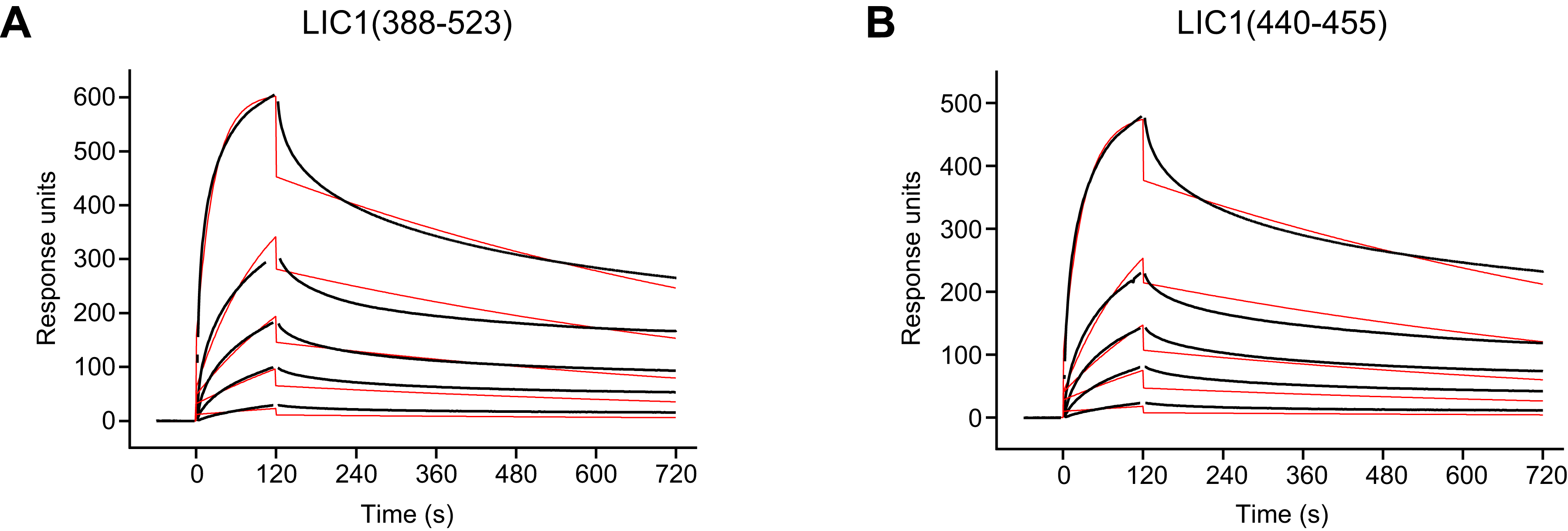

Supplement: S6 Fig — (A, B) Sensogram (black lines) and corresponding 1:1 fitting (red lines) of the interaction between GST::LIC1(388–523)::6xHis (A) or GST::LIC1(440–455)::6xHis (B) with RILP::Strep-tag II. One of three replicates is shown. GST::LIC1 constructs were immobilized on the sensor chip with an anti-GST antibody. Fitted constants (mean ± SEM): Ka = 8.72 ± 0.83 × 10^2^ M−1 s−1 and Kd = 10.10 ± 0.46 × 10−4 s−1, which results in KD = 1.18 ± 0.14 × 10^−6^ M for LIC1(388–523) (A); Ka = 6.06 ± 0.31 × 10^2^ M−1 s−1 and Kd = 9.96 ± 0.72 × 10−4 s−1, which results in KD = 1.67 ± 0.21 × 10^−6^ M for LIC1(440–455) (B). Underlying data for S6 Fig can be found in S1 Data. BICD2, bicaudal D homolog 2; GST, glutathione S-transferase; LIC 1, light intermediate chain 1; RILP, RAB-interacting lysosomal protein. (TIF) [file pbio.3000100.s006.tif]

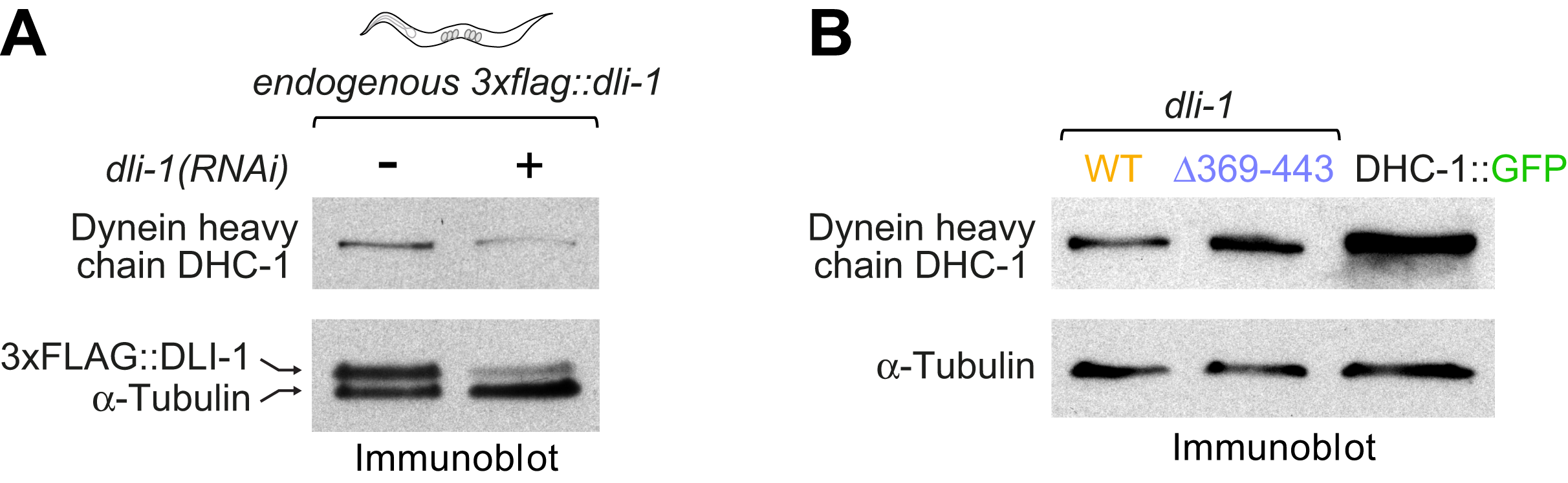

Supplement: S7 Fig — (A) Immunoblot of adult hermaphrodites expressing endogenously tagged 3xFLAG::DLI-1, showing that depletion of DLI-1 by RNAi reduces DHC-1 levels. α-Tubulin serves as the loading control. (B) Immunoblot comparing DHC-1 levels in wild-type and dli-1(Δ369–443) animals with those in animals expressing endogenous DHC-1 tagged with GFP. α-Tubulin serves as the loading control. Note that GFP-tagged DHC-1 is expressed at higher levels than DHC-1 in either wild-type or dli-1(Δ369–443) animals. Since DHC-1::GFP animals do not exhibit any obvious defects [37], the slight increase in DHC-1 levels in dli-1 mutants relative to wild-type animals is unlikely to be the cause for the phenotype. DHC-1, dynein heavy chain 1; GFP, green fluorescent protein; RNAi, RNA interference. (TIF) [file pbio.3000100.s007.tif]

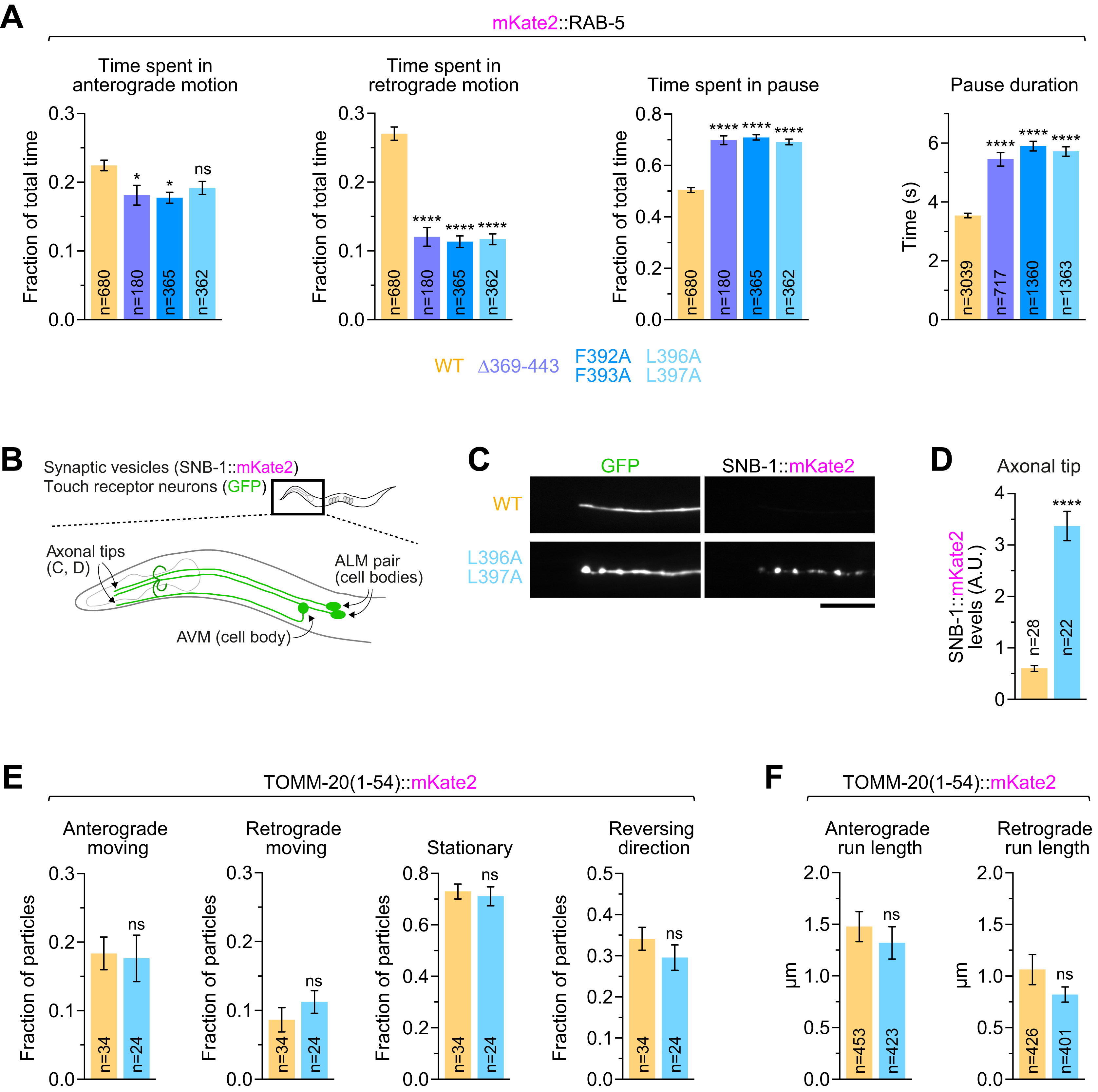

Supplement: S8 Fig — (A) Quantification of early endosome motility (mKate2::RAB-5), based on the analysis of kymographs as shown in Fig 5F. Graphs represent the mean ± SEM. For time spent in anterograde motion, retrograde motion, or pause, n represents the total number of tracks. For pause duration, n reflects the total number of segments within tracks of moving particles framed by a pause or a reversal. Results are derived from 2–5 independent imaging sessions. Statistical significance (mutant versus WT dli-1) was determined by one-way ANOVA on ranks (Kruskal-Wallis nonparametric test) followed by Dunn's multiple comparison test. ****P < 0.0001; *P < 0.05; ns indicates P > 0.05. (B) Cartoon showing the axonal tips imaged in animals coexpressing the synaptic vesicle marker SNB-1::mKate2 and soluble GFP in touch receptor neurons. (C) Fluorescence images of axonal tips in day 1 adults, showing misaccumulation of synaptic vesicles in the dli-1(L396A/L397A) mutant. Scale bar, 10 μm. (D) Quantification of synaptic vesicle misaccumulation in axonal tips using fluorescence intensity measurements of SNB-1::mKate2 as shown in (C). Graph represents the mean ± SEM signal in A.U. for n number of neurons imaged in two independent experiments. Statistical significance was determined with the Mann-Whitney test. ****P < 0.0001. (E, F) Quantification of mitochondrial motility (TOMM-20[1–54]::mKate2), based on the analysis of kymographs as shown in Fig 7F. Graphs represent the mean ± SEM. The total number n of axons (E) or segments (F) is indicated. Results are derived from 2–5 independent imaging sessions. Statistical significance was determined with the Mann-Whitney test. ns indicates P > 0.05. Underlying data for S8 Fig can be found in S1 Data. A.U., arbitrary units; GFP, green fluorescent protein; ns, not significant; SNB-1, synaptobrevin 1; WT, wild type. (TIF) [file pbio.3000100.s008.tif]
