# Supplementary material for: A transient helix in the disordered region of dynein light intermediate chain links the motor to structurally diverse adaptors for cargo transport
Source: PLoS Biol. 2019 Jan 7;17(1):e3000100. doi: 10.1371/journal.pbio.3000100 (PMC6336354; doi:10.1371/journal.pbio.3000100)
Supplement: S1 Table — (DOCX) [file pbio.3000100.s011.docx]

| Strain | Genotype |
| --- | --- |
| N2 | WT (ancestral N2 Bristol) |
| GCP66 | *nud-2(ok949) I* |
| GCP302 | *unc-119(ed3) III?; prtSi99[pRG525; Pmec-7::tomm-20(aa1-54)::mKate2::tbb-2 3'UTR; cb-unc-119(+)] II; zdIs5[Pmec-4::gfp; lin-15(+)] I ** |
| GCP311 | *unc-119(ed3) III?; prtSi104[pRG527; Pmec-7::mKate2::rab-5::tbb-2 3'UTR; cb-unc-119(+)] II; zdIs5[Pmec-4::gfp; lin-15(+)] I ** |
| GCP330 | *unc-119(ed3) III?; prtSi101[pRG526; Pmec-7::snb-1::mKate2::tbb-2 3'UTR; cb-unc-119(+)] II; zdIs5[Pmec-4::gfp; lin-15(+)] I ** |
| GCP611 | *dli-1[prt96(Δaa369-443)] IV; IV/nT1[qls51];V/nT1[qls51](IV/V)* |
| GCP639 | *dli-1[prt73(3xflag::dli-1)] IV* |
| GCP671 | *dli-1[prt106(F392A/F393A)] IV; IV/nT1[qls51];V/nT1[qls51](IV/V)* |
| GCP672 | *dli-1[prt107(L396A/L397A)] IV; IV/nT1[qls51];V/nT1[qls51](IV/V)* |
| GCP673 | *dli-1[prt96(Δaa369-443)] IV; IV/nT1[qls51];V/nT1[qls51](IV/V); unc-119(ed3) III?; prtSi104[pRG527; Pmec-7::mKate2::rab-5::tbb-2 3'UTR; cb-unc-119(+)] II; zdIs5[Pmec-4::gfp; lin-15(+)] I ** |
| GCP679 | *dli-1[prt106(F392A/F393A)] IV; IV/nT1[qls51];V/nT1[qls51](IV/V); unc-119(ed3) III?; prtSi104[pRG527; Pmec-7::mKate2::rab-5::tbb-2 3'UTR; cb-unc-119(+)] II; zdIs5[Pmec-4::gfp; lin-15(+)] I ** |
| GCP680 | *dli-1[prt107(L396A/L397A)] IV; IV/nT1[qls51];V/nT1[qls51](IV/V); unc-119(ed3) III?; prtSi104[pRG527; Pmec-7::mKate2::rab-5::tbb-2 3'UTR; cb-unc-119(+)] II; zdIs5[Pmec-4::gfp; lin-15(+)] I ** |
| GCP696 | *dli-1[prt107(L396A/L397A)] IV; IV/nT1[qls51];V/nT1[qls51](IV/V); unc-119(ed3) III?; prtSi101[pRG526; Pmec-7::snb-1::mKate2::tbb-2 3'UTR; cb-unc-119(+)] II; zdIs5[Pmec-4::gfp; lin-15(+)] I ** |
| GCP697 | *dli-1[prt107(L396A/L397A)] IV; IV/nT1[qls51];V/nT1[qls51](IV/V); unc-119(ed3) III?; prtSi99[pRG525; Pmec-7::tomm-20(aa1-54)::mKate2::tbb-2 3'UTR; cb-unc-119(+)] II; zdIs5[Pmec-4::gfp; lin-15(+)] I ** |
| GCP702 | *dli-1[prt111(Δaa414-443)] IV* |
| GCP703 | *dli-1[prt111(Δaa414-443)] IV; unc-119(ed3) III?; ijmSi8[pJD362; Pmex-5::gfp::tbb-2 3'UTR; mCherry::his-11; cb-unc-119(+)] II ** |
| GCP713 | *dli-1[prt111(Δaa414-443)]IV; unc-119(ed3) III?; prtSi104[pRG527; Pmec-7::mKate2::rab-5::tbb-2 3'UTR; cb-unc-119(+)] II; zdIs5 [Pmec-4::gfp; lin-15(+)] I ** |
| GCP724 | *dli-1[prt111(Δaa414-443)] IV; nud-2(ok949) I* |
| GCP728 | *nud-2(ok949) I; unc-119(ed3) III?; ijmSi8[pJD362[Pmex-5::gfp::tbb-2 3'UTR; mCherry::his-11; cb-unc-119(+)] II ** |
| GCP729 | *dli-1[prt111(Δaa414-443)] IV; nud-2(ok949) I; unc-119(ed3) III?; ijmSi8[pJD362; Pmex-5::gfp::tbb-2 3' UTR; mCherry::his-11; cb-unc-119(+)] II ** |
| GCP759 | *dli-1[prt96(Δaa369-443)] IV; IV/nT1[qls51];V/nT1[qls51](IV/V); unc-119(ed3) III?; prtSi99[pRG525; Pmec-7::tomm-20(aa 1-54)::mKate2::tbb-2 3'UTR; cb-unc-119(+)] II; zdIs5[Pmec-4::gfp; lin-15(+)] I** |
| JDU21 | *unc-119(ed3) III; ijmSi8[pJD362; Pmex-5::gfp::tbb-2 3'UTR; mCherry::his-11; cb-unc-119(+)] II* |
| OD2955 | *dhc-1[lt45(dhc-1::gfp)] I* |

* *[unc-119(ed3) III?]* was present in the paternal strains, but these strains have not been directly sequenced to determine if the ed3 mutation is present in the *unc-119* gene.
