# Supplementary material for: A transient helix in the disordered region of dynein light intermediate chain links the motor to structurally diverse adaptors for cargo transport
Source: PLoS Biol. 2019 Jan 7;17(1):e3000100. doi: 10.1371/journal.pbio.3000100 (PMC6336354; doi:10.1371/journal.pbio.3000100)
Supplement: S2 Table — CRISPR/Cas9, clustered regularly interspaced short palindromic repeat/CRISPR-associated 9; sgRNA, single guide RNA. (DOCX) [file pbio.3000100.s012.docx]

| Gene ID | Gene Name | Modification | sgRNA and PAM motif |
| --- | --- | --- | --- |
| C39E9.14 | *dli-1* | N-terminal 3xFLAG | CCAACTGCGCAACCACTGGTGG |
| C39E9.14 | *dli-1* | Δ369-443 | CTGGCAACTTCAACCACTACGG  GTTATGCATCACTGTCCCGGGG |
| C39E9.14 | *dli-1* | F392A/F393A | TGGAGAAGAAATTGGCGAGTGG  AGCAAGTTGGAGAAGAAATTGG |
| C39E9.14 | *dli-1* | L396A/L397A | TTTACCTTATTGAGCAAGTTGG  TTCTCCAACTTGCTCAATAAGG |
| C39E9.14 | *dli-1* | Δ414-443 | TCCACTGTTTCCCAGCCGATGG  GCTGCGGCATCCATCGGCTGGG  GTTATGCATCACTGTCCCGGGG |
